# Biobased carbon dots as photoreductants – an investigation by using triarylsulfonium salts

**DOI:** 10.3762/bjoc.21.84

**Published:** 2025-05-26

**Authors:** Valentina Benazzi, Arianna Bini, Ilaria Bertuol, Mariangela Novello, Federica Baldi, Matteo Hoch, Alvise Perosa, Stefano Protti

**Affiliations:** 1 Department of Chemistry, University of Pavia, Viale Taramelli 12, 27100 Pavia, Italy; 2 Department of Molecular Sciences and Nanosystems, Università Ca’ Foscari Venezia, Via Torino 155, 30172 Venezia Mestre, Italy; 3 Department of Chemistry, Life Sciences and Environmental Sustainability, Università di Parma, Parco Area delle Scienze 17/A, 43124 Parma, Italy

**Keywords:** agricultural waste, carbon dots (CDs), triarylsulfonium salts, visible light

## Abstract

We investigated the potential application of six types of carbon dots (CDs) obtained from different organic sources as photoreductants. Such carbon nanomaterials were synthesized by two different approaches, either hydrothermal or pyrolytic, from citric acid and glucose as the starting organic substrates. On the other hand, carbon dots deriving from fishery waste (bass scales) and fruit processing waste (blackberries) have been also prepared. Diethylenetriamine was employed in some cases as the nitrogen source. The hydrothermal syntheses yielded amorphous CDs, which were either non-doped (a-CDs) or nitrogen-doped (a-N-CDs), whereas the pyrolytic treatment afforded graphitic CDs (g-CDs). The efficiency of the so obtained carbon nanomaterials was studied in the model photoreduction reaction of triarylsulfonium salts to diaryl sulfides. A comparison carried out on the results obtained points out the key role of the starting substrates in determining the photophysics and the photochemical efficiency of the resulting CDs. In this context, citric acid-derived materials (both graphitic and amorphous) were found as the most promising materials, while less satisfactory results have been observed when using CDs derived from glucose and biowastes.

## Introduction

Carbon dots (CDs) are a class of zero-dimensional carbon-based semiconducting nanoparticles bearing on the surface a wide range of functional groups, such as carboxylic acids, alcohols, and amines, that garnered significant attention in the last decade among the carbon nanomaterials category in view of their stability, water affinity/dispersibility, and low toxicity [1–4]. Such nanomaterials significantly absorb in the 280–350 nm region due to a wide range of π–π* (C=C) and n–π* (C=O) transitions in both the core and on the surface of the particles. CDs typically exhibit excitation-dependent emission spectra and large Stokes shifts, features that are uncommon in traditional molecular fluorophores and semiconductor nanoparticles, and such behavior is attributed to the intrinsic heterogeneity of the CDs. The peculiar photophysics of carbon dots arises from their size, surface defects, and functional groups, which can be engineered to tune their optical properties. Accordingly, their emission spans a broad range of wavelengths, from ultraviolet to the visible region, allowing for the potential application in an impressive range of applications, such as, among the others, optoelectronics [5], sensing [6] and biomedicine (including bioimaging [7] and drug delivery [8]).

Unlike traditional semiconductors, CDs are composed primarily of carbon, which makes them environmentally friendly easy-to-synthesize and appealing nanomaterials for photocatalysis [9] and photopolymerization [10–12].

For these reasons, several strategies starting from different carbon-based materials have been proposed and optimized to prepare CDs. In general, such methods are classified in two categories, namely “top-down” and “bottom-up” [1–4,13]. In the top-down approach, CDs arise from the decomposition of large carbogenic structures (including graphite, graphite sheets and carbon nanotubes), whereas in the bottom-up approach such materials are obtained by carbonization of small organic molecules and biowastes (carbohydrates, polymers, bioorganic compounds, organosilanes). The latter strategy is the most prevalent one, due to the simplicity of the procedures, environmental friendliness, and equipment required, as well as the ability to use a large variety of precursors. In the bottom-up synthesis methods, conversion of the carbon precursors is obtained by having recourse to treatments such as pyrolysis, ultrasonic carbonization, hydrothermal, solvothermal, or microwave irradiation [13–17].

**Scheme 1:**
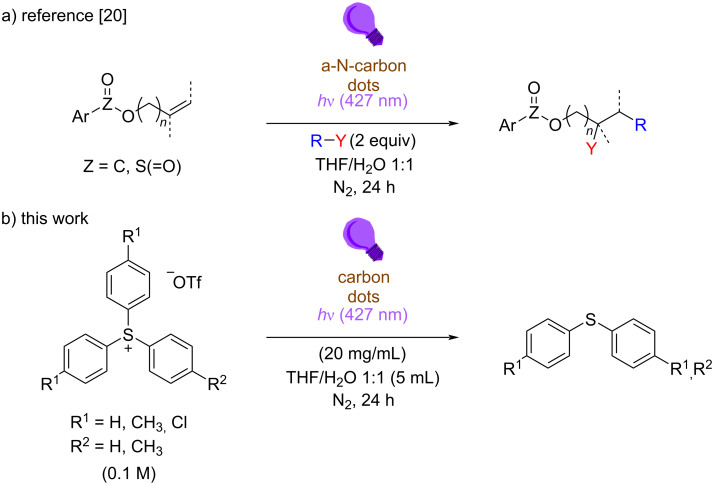
a) CDs-mediated 1,2-difunctionalization of alkenes by alkyl halides R–Y and b) light-driven reduction of triarylsulfonium salts in the presence of CDs.

The properties of CDs can be tuned by doping them with various elements, which influence photophysical and electrochemical properties, stability, and biocompatibility. The electrochemical properties of such materials have been then evaluated by cyclic voltammetry (CV). For all the properties mentioned above, CDs emerged as low-cost and sustainable photocatalysts. Indeed, upon visible-light irradiation, the generated excited state CD* can operate as either photooxidant or photoreductant in the presence of a suitable electron donor or acceptor [18], and these properties have been exploited in procedures for the formation of both C–C and C–heteroatom bonds [18–19]. Our research groups recently focused on the application of CDs in light-mediated organic synthesis, with particular attention to the 1,2-difunctionalization of olefins by alkyl halides via atom transfer radical addition processes in the presence of amorphous nitrogen-doped carbon dots (Scheme 1a) [20]. In the framework of this research topic, we decided to investigate the use of carbon dots as photoreductants, and we considered triarylsulfonium salts Ar_3_S^+^X^−^ as suitable probes. Such derivatives are photoreactive molecules characterized by a positively charged sulfur atom bonded to three aromatic moieties [20–23], mainly employed as photoacid generators in cationic polymerization [19]. Apart from direct photoreactivity [24–25], sulfonium salts can be easily reduced under photoredox-catalyzed conditions [20–22], and the resulting radical undergoes homolytic cleavage of one of the C–S bonds, releasing an aryl radical Ar^•^ and a diaryl sulfide Ar_2_S. Subsequently, triarylsulfonium ions have been considered as a promising source of aryl radicals and employed in organic synthesis [20–23,26–32].

This investigation aims to compare the performance of CDs prepared from several carbon precursors including citric acid, glucose, and organic waste materials via different chemical treatments (hydrothermal and pyrolytic methods). The effect of nitrogen doping on the performance of the examined CDs has been also investigated.

## Results and Discussion

**Synthesis, photophysical and electrochemical properties of CDs.** As hinted above, CDs have been prepared from different carbon precursors, including citric acid, glucose, and two kinds of biowaste, namely bass scales and blackberry residues arising from the production of preserves and jams and furnished by Rigoni di Asiago s.r.l. The strategy employed for the preparation of CDs were pyrolysis and hydrothermal treatment; the first one involves the direct carbonization (at 200–400 °C) of the precursor material for 24–100 hours under solvent-free conditions. The obtained carbon nanomaterials exhibit predominantly a graphitic core structure. In contrast, under hydrothermal conditions, the starting substrates were dissolved or suspended in water, and the resulting mixture transferred to an autoclave and treated at a temperature ranging 140–200 °C; the treatment yields CDs with an amorphous structure. The synthetic procedure, as well as the structural and morphological characteristics of these materials, have been previously reported by the research group of M. Selva and A. Perosa (see also Supporting Information File 1 for further details) [30–35].

**Figure 1:**
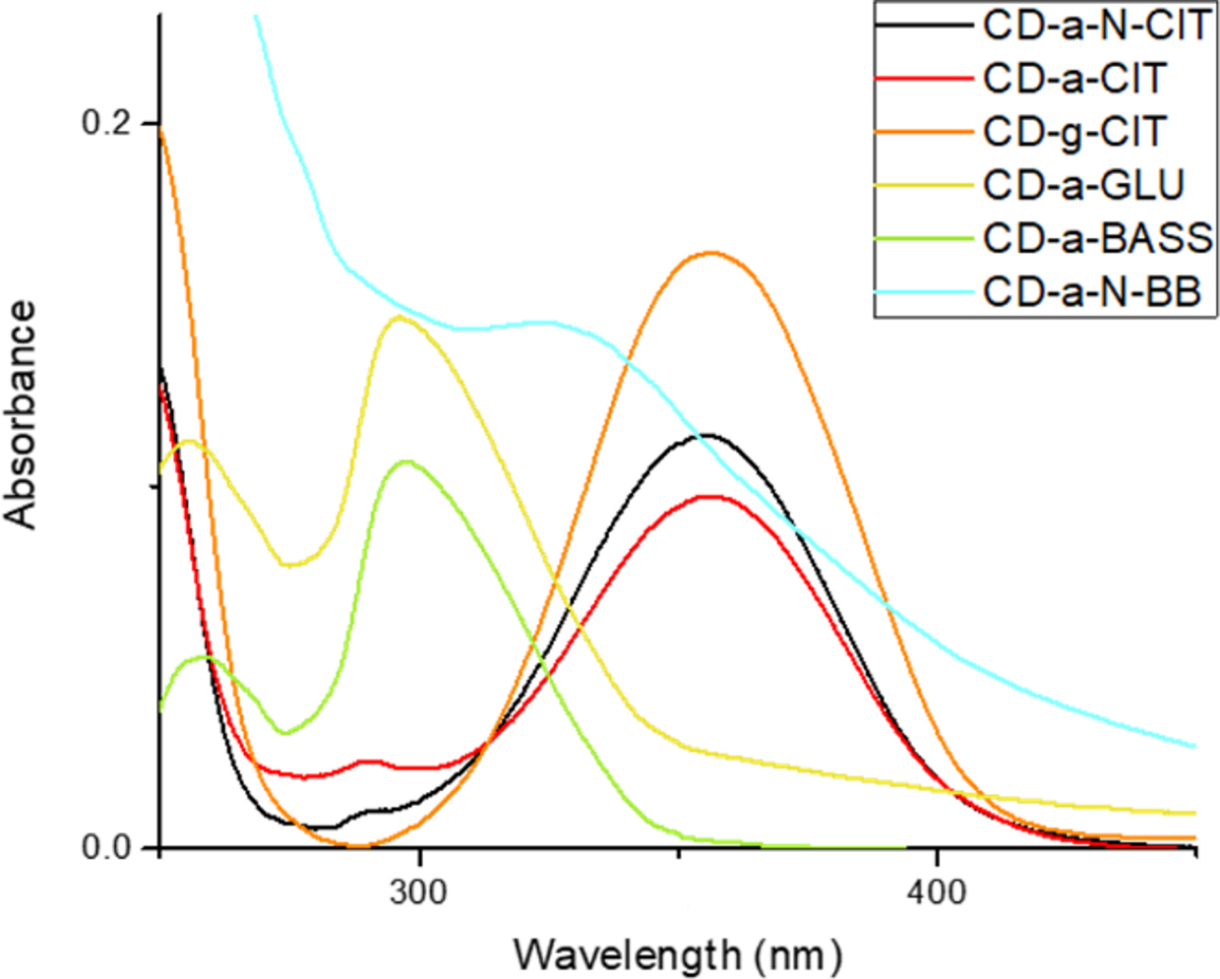
UV–vis spectra of the CDs. All the measurements have been performed in water, except for CD-a-GLU, where a H_2_O/acetonitrile 1:1 solution was examined, for solubility reasons.

As shown in Table 1, the materials synthesized via pyrolysis exhibit a graphitic core, characterized by an ordered structure, whereas those synthesized via hydrothermal treatments have an amorphous structure. The optical properties of the prepared CDs were thus investigated by UV–vis absorption spectroscopy. The obtained spectra (Figure 1) showed three main absorption bands located at 225–235 nm (assigned to, according to the literature, the π–π* transition of the sp^2^ C=C conjugated system), 265–280 nm (in turn ascribed to the n–π* transition of the C=O group on the surface), and 350–360 nm (due to the n–π* transition of defects states). The spectra of both doped and undoped CDs obtained from citric acid are mainly comparable (see Figure 1), with a predominant band located at 350 nm, CDs synthesized from glucose and bass scales show a significant absorption at around 260 nm and 300 nm, respectively [33–35]. A different UV–vis spectrum was obtained for the Blackberries-derived nitrogen-doped material, which showed a main absorption at 330 nm, with a tail extending to the visible region.

**Table 1:** Carbon dots tested in this project and their main (photo)physical characteristics.

Entry	Carbon dot	Precursor	Doping	Approach	Core structure

1	CD-a-N-CIT	citric acid	DETA^a^	hydrothermal	amorphous
2	CD-a-CIT	citric acid	–	hydrothermal	amorphous
3	CD-g-CIT	citric acid	–	pyrolysis	graphitic
4	CD-a-GLU	glucose	–	hydrothermal	amorphous
5	CD-a-BASS	bass scale	–	hydrothermal	amorphous
6	CD-a-N-BB	blackberries	DETA^a^	hydrothermal	amorphous

^a^DETA: diethylenetriamine, see Supporting Information File 1 for further details.

The emission properties of the synthesized CDs were also investigated and the results are summarized in Table 2. The fluorescence quantum yield strongly depends on the nature of the starting substrates, as is apparent by comparison of the value obtained for graphitic citric acid-derived carbon dots CD-g-CIT (Φ_F_ = 0.015) and the value for the amorphous citric acid derivative CD-a-CIT (Φ_F_ = 0.74). The measured emission lifetime is also dependent on the nature of the materials, ranging from 2.4 to 13 ns, with a biexponential decay (see Supporting Information File 1 for CD-a-N-BB).

**Table 2:** Emission parameters for the examined CDs.

Carbon dots	λ_em_ (nm)	Φ_F_^a^	τ (ns)

CD-a-N-CIT	439	0.51	13.0
CD-a-CIT	439	0.74	3.6
CD-g-CIT	458	0.015	4.3
CD-a-GLU	357	0.62	4.6
CD-a-BASS	407	0.067	2.4
CD-a-N-BB	443	0.080	3.4

^a^Quinine sulfate (Φ_F_ = 0.546 in H_2_SO_4_ 0.5 M) used as reference [36].

**Light-mediated reduction of triarylsulfonium salts.** At this point, a Stern–Volmer analysis in the presence of tri-*p*-tolylsulfonium triflate (**1b**) as the model substrate was employed to quantify the quenching rate coefficient (*K*_q_), in order to determine the interaction between the four different CDs from citric acid and from blackberries with triarylsulfonium salts. A diffusional rate value was found for all examined nanomaterials (Table 3), pointing to an efficient interaction between the photoexcited CDs and **1b**.

**Table 3:** Quenching constants of the CDs calculated on the fluorescence intensity.

Carbon dots	*k*_q_(*I*) (×10^10^ M^−1^ s^–1^)

CD-a-N-CIT	2.14
CD-a-CIT	9.78
CD-g-CIT	16.00
CD-a-N-BB	1.70

Cyclic voltammetry experiments have been carried out on both CDs (whose *E*_REDonset_ value were in the range −1.60 and −1.92 V vs Ag/AgCl, a set comparable to that reported in the literature for *fac*-Ir(ppy) [37], which was effectively used in the photoredox-catalyzed reduction of sulfonium salts [29]) and derivatives **1a**–**d** (*E*_Ar3S_^+^_/Ar3S_^•^ = −1.35 to −1.46 V vs Ag/AgCl; see Supporting Information File 1 for further information). The obtained results highlighted the feasibility of the reduction process, thus, a nitrogen-saturated solution of **1a**–**d** (0.1 M) in THF/H_2_O 1:1 in the presence of the CDs was irradiated at 427 nm for 24 h and the photolyzed solution analyzed.

As summarized in Table 4, the results obtained with amorphous CDs derived from citric acid are almost comparable and satisfactory (see for instances Table 4, entries 1, 2, and 7) with the only exception of salt **1b** (Table 4, entry 10), that was poorly consumed in the presence of CD-g-CIT. On the other hand, CDs derived from glucose, as well as those obtained from the treatment of biowaste (bass scales and blackberries) were poorly performant, and the yield of the expected diary sulfides did not exceed 12% (see for instance Table 4, entries 9, 11, and 12). In the presence of unsymmetrically substituted triarylsulfonium salts **1c** and **d**, the reduction afforded a mixture of diphenyl sulfide (**2a**) and **2c**/**d**, with the monosubstituted ones slightly favored. Finally, a solution of triphenylsulfonium triflate (**1a**) was irradiated under the same reaction conditions in the absence of CDs, and no reaction of the starting material was observed, thus pointing out the key role of CDs in the process. Some additional irradiation was carried out to identify the aryl radical released during the irradiation. Unfortunately, when the reaction was conducted in the presence of both furan and allyl phenyl sulfone [27] no arylation product was detected.

**Table 4:** Light-mediated reduction of triarylsulfonium salts in the presence of the CDs examined in this work.

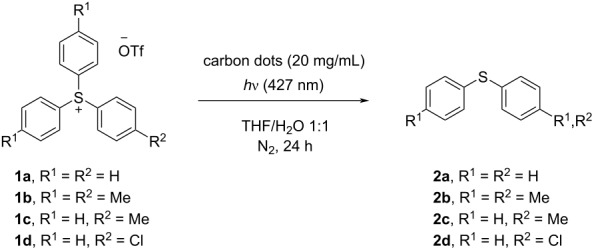			

Entry	Ar_3_S^+^	CDs	products, % yield

1	**1a**	CD-a-N-CIT	**2a**, 42^a^
2		CD-a-CIT	**2a**, 54^b^
3		CD-a-GLU	**2a**, 7^b^
4		CD-g-CIT	**2a**, 55^b^
5		CD-a-BASS	**2a**, 7^b^
6		CD-a-N-BB	**2a**, 10^b^

7	**1b**	CD-a-N-CIT	**2b**, 69^a^
8		CD-a-CIT	**2b**, 61^b^
9		CD-a-GLU	**2b**, 9^b^
10		CD-g-CIT	**2b**, 33^b^
11		CD-a-BASS	**2b**, 3^b^
12		CD-a-N-BB	**2b**, 11^b^

13	**1c**	CD-a-N-CIT	**2a**, 9^b^, **2c**, 37^b^
14		CD-a-CIT	**2a**, 14^a^, **2c**, 33^a^
15		CD-a-GLU	**2a**, 3^b^, **2c**, 4^b^
16		CD-g-CIT	**2a**, 14^b^, **2c**, 31^b^
17		CD-a-BASS	**2a**, 4^b^, **2c**, 6^b^
18		CD-a-N-BB	**2a**, 7^b^, **2c**, 10^b^

19	**1d**	CD-a-N-CIT	**2a**, 36^a^, **2d**, 43^a^
20		CD-a-CIT	**2a**, 27^b^, **2d**, 30^b^
21		CD-a-GLU	**2a**, 5^b^, **2d**, 5^b^
22		CD-g-CIT	**2a**, 33^b^, **2d**, 33^b^
23		CD-a-BASS	**2a**, 11^b^, **2d**, 12^b^
24		CD-a-N-BB	**2a**, 12^b^, **2d**, 12^b^

^a^Isolated yields; ^b^GC yields quantified by using dodecane as internal standard.

## Conclusion

The results arising from cyclic voltammetry analyses, along with the Stern–Volmer fluorescence quenching data and with the isolated products and the GC yields obtained when irradiating the carbon dots in the presence of triarylsulfonium salts, indicate that carbon dots are effective in the photoreduction of triarylsulfonium salts. However, the nature of the starting material used to prepare the CDs is the most significant parameter influencing both the photophysical and photochemical properties of such carbon nanomaterials. Those obtained from citric acid were found to be the most promising catalysts in photoreduction, regardless of the fact that they are amorphous or graphitic. Comparable results were obtained from both undoped and nitrogen-doped citric acid derived CDs (compare for instance results depicted in entries 1, 2, 4 in Table 4). On the other hand, carbon nanomaterials synthesized from glucose, as well as those prepared starting from food wastes (bass scales and black berry residues from fruit preserves) resulted poorly efficient in the examined process.

The obtained results also pointed out the efficiency of citric acid-derived CDs in the photoreduction of Ar_3_S^+^ species, a process where transition-metal-based complexes are employed [29], often (in the case of Ru(II)-based derivatives) in the presence of tertiary amines as sacrificial reductants [27–28]. Nonetheless, while this work serves primarily as a preliminary study comparing the reducing power of various types of CDs and aims to promote the use of these sustainable materials, it opens the way for further exploitation of such compounds in visible-light-catalyzed reactions.

## Supporting Information

File 1 Experimental details, photophysics of CDs, cyclic voltammetry of triarylsulfonium salts and NMR spectra.

## Data Availability

All data that supports the findings of this study is available in the published article and/or the supporting information of this article.
