# Supplementary material for: Biobased carbon dots as photoreductants – an investigation by using triarylsulfonium salts
Source: Beilstein J Org Chem. 2025 May 26;21:1024–30. doi: 10.3762/bjoc.21.84 (PMC12130624; doi:10.3762/bjoc.21.84)

BEILSTEIN JOURNAL OF ORGANIC CHEMISTRY

Supporting Information

for

Biobased carbon dots as photoreductants – an investigation by using triarylsulfonium salts

Valentina Benazzi, Arianna Bini, Ilaria Bertuol, Mariangela Novello, Federica Baldi, Matteo Hoch, Alvise Perosa and Stefano Protti

Beilstein J. Org. Chem. **2025,** *21,* 1024–1030. doi:10.3762/bjoc.21.84

Experimental details, photophysics of CDs, cyclic voltammetry of triarylsulfonium salts and NMR spectra

License and Terms: This is a supporting information file under the terms of the Creative Commons Attribution License (https://creativecommons.org/licenses/by/4.0). Please note that the reuse, redistribution and reproduction in particular requires that the author(s) and source are credited and that individual graphics may be subject to special legal provisions.
The license is subject to the *Beilstein Journal of Organic Chemistry* terms and conditions: (https://www.beilstein-journals.org/bjoc/terms)

Table of contents

1. Experimental part S2
   1.1 General information S2
   1.2 Preparation of carbon dots (CDs) employed in the present work S3
   1.3 Synthesis of triarylsulfonium salts **1** S6
   1.4 Light-mediated reduction of triarylsulfonium salts **1a**–**d** S8
   1.5 Photophysics of CDs S13
   1.6 Cyclic voltammetry of triarylsulfonium salts S18
2. References S21
3. Copies of ^{1}H and ^{13}C NMR spectra S22

1. Experimental part

1.1 General information

All reagents, chemicals, and solvents used in this work were sourced from various commercial suppliers (TCI Europe, Merck - Sigma Aldrich, Fluorochem, Carlo Erba). Solvents were used as supplied without any further purification. THF was distilled prior to use. The carbon dots used as photocatalyst (named CDs) were provided by the Department of Molecular Sciences and Nanosystems of the Ca' Foscari University of Venice and prepared according to the procedures shown in the main text and described in detail in the bibliographic reference indicated. NMR spectra were recorded on a 300 (for ^{1}H) or 75 (for ^{13}C) MHz spectrometer; the attributions were made based on ^{1}H and ^{13}C NMR. Data for ^{1}H NMR are reported as follows: chemical shift referred to TMS (δ ppm), multiplicity (s = singlet, bs = broad singlet, d = doublet, t = triplet, q = quadruplet, quint = quintuplet, sext = sextuplet, sept = septuplet, m = multiplet), coupling constant (Hz) and integration. Data for ^{13}C NMR are reported in terms of chemical shift.

The light source for photochemical experiments was a 40 W Kessil LED (centered at 427) at full power (technical specifications at https://www.kessil.com/science/PR160L). Thin-layer chromatography (TLC) was performed on silica gel 60 F-254 plates. Visualization of the developed plates was performed by fluorescence quenching or $KMnO_4$ staining. Column chromatography was carried out using Sigma Aldrich silica gel 60 (70–230 mesh).

GC-FID analyses were performed on an Agilent 7820A instrument. The injection was performed at 250 °C in split mode. The initial oven temperature of 80 °C was maintained for 2 min, increased by 10 °C min^{-1} to 250 °C and held for 5 min. An Agilent HP5 30 m × 0.32 mm × 0.25 µm film thickness capillary column was used with nitrogen as the carrier gas at a constant flow rate of 6.0 mL min^{-1}.

GC/MS analyses were carried out on a Thermo Scientific DSQII single quadrupole GC/MS system (TraceDSQII mass spectrometer, Trace GC Ultra gas chromatograph, TriPlus autosampler - ThermoFisher Scientific, Waltham, MA, USA). Chromatography was performed on a Rxi-5Sil MS capillary column (30 m length × 0.25 mm ID × 0.25 µm film thickness, Restek, Milan, Italy) with Helium (>99.99%) as carrier gas at a constant flow-rate of 1.0 mL min^{-1}. An injection volume of 1 µL was employed. The injector temperature was set at 250 °C and it was operated in split mode, with a split flow of 10 mL/min. The oven temperature was programmed from 80 °C (isothermal for 2 min) to 220 °C at the rate of 10 °C/min, then from 220 °C to 300 °C (isothermal for 5 min) at the rate of 4 °C/min. Mass transfer line temperature was set at 260 °C. Total GC running time was 41 min. All mass spectra were acquired with an electron ionization system (EI, electron impact mode) with

ionization energy of 70 eV and source temperature of 250 °C, with spectral acquisition in full scan mode, positive polarity, over a mass range of 35–650 Da with a scan rate of 940 amu/s. The chromatogram acquisition, detection of mass spectral peaks and their waveform processing were performed using Xcalibur MS Software Version 2.1 (Thermo Scientific Inc.). Assignment of chemical structures to chromatographic peaks was based on the comparison with the databases for GC-MS NIST Mass Spectral Library (NIST 08) and Wiley Registry of Mass Spectral Data (8th edition).

Cyclic voltammetry of Ar_3S^+ TfO^- salts was carried out by means of a Amel model 4330 module equipped with a 5 mL standard three-electrode cell with a glassy carbon (0.49 cm^2 geometrical area) working electrode, a platinum wire as auxiliary electrode and an Ag/AgCl, 3 M NaCl reference electrode, all obtained from BASi Electrochemistry. A solution of DMF and 200 µL of water containing 0.1 M lithium perchlorate was used as solvent and supporting electrolyte, scanning the potential in the range from 0 to −2400 mV, with a 5 mM compound concentration and a scan speed of 100 mVs^{-1}. At this point, two of the four salts used were synthesized (because the others were commercially available), through a reaction between a differently substituted diphenyl sulfoxide and a chosen aryl. UV–vis absorption spectra were recorded on a V-550 Jasco spectrophotometer. Fluorescence spectra were performed using a LS-55 Perkin Elmer spectrofluorometer. Transmission electron microscopy images were collected on a Jeol JEM-1200 EX II instrument. 10 µL of the colloidal sample were dropped on nickel grids, 300 mesh, coated with Collodion solution. Photoluminescence measurements were carried out by means of a FLS1000 Edinburgh Fluorometer.

1.2 Preparation of carbon dots (CDs) employed in the present work

The CDs examined in the present investigation have been synthesized in Cà Foscari University (Venice) in the research group of Selva and Perosa[S1-S4] by adopting the procedures described in the following. Where not included in the present section, the characterization data have been described in refs. S1–S4.

a) *Synthesis of* CD-a-N-CIT

The nanomaterials were synthesized under hydrothermal conditions by heating an aqueous solution of citric acid (2 g, 10.41 mmol in 20 mL of MilliQ water) and diethylenetriamine (0.67 g, 6.49 mmol) in a sealed autoclave at 180 °C for 6 h. The mixture was then evaporated to dryness leading to a brown solid with 72 wt % yield that was used without any further purification.[S1,S2]

b) Synthesis of CD-a-CIT

The nanomaterials were synthesized under hydrothermal conditions by heating an aqueous solution of citric acid (2 g, 10.41 mmol in 20 mL of MilliQ water) in a sealed autoclave at 180 °C for 24 h. The mixture was then neutralized to pH 7 with aqueous NaOH and evaporated to dryness leading to a dark yellow oil with 25 wt % yield that was employed without any further purification.[S1,S2]

c) Synthesis of CD-g-CIT

The graphitic nanomaterials were synthetized by pyrolysis, heating neat citric acid at 220 °C for 48 h in a conical flask equipped with a distillation apparatus. The resulting crude solid was then neutralized to pH 7 with aqueous NaOH and then dialyzed against milliQ water (2 L) for 24 h using a 1 kDa MWCO dialysis bag, changing the water every 12 h. The inner solution was evaporated to dryness leading to a dark-black solid with 20 wt % yield.[S1,S2]

d) Synthesis of CD-a-GLU

The nanomaterials have been prepared under hydrothermal conditions. 2.0 g (11.10 mmol) of glucose were dissolved in 20 mL of MilliQ water until a clear solution was obtained. The mixture was then placed in a glass reactor, which was sealed inside a stainless-steel autoclave; the autoclave was heated at 200 °C for 24 h and then allowed to cool to room temperature. The mixture was centrifuged at 6000 rpm to remove the black precipitate, and the supernatant was filtered through a 0.22 μm syringe filter. Water was removed first by rotary evaporation and then by heating at 120 °C under high vacuum to obtain a black solid with 34% yield.[S3]

e) Synthesis of CD-a-BASS

The scales of sea bass (*Dicentrarchus labrax*) were purchased from a local market. Prior to use, the scales were washed thoroughly with water and dried in a vacuum oven at 70 °C overnight. In a typical procedure, 2 g of fish scales were put in a Teflon-lined autoclave with 20 mL of MilliQ water and heated at 200 °C for 24 h. The obtained brownish suspension was filtered and evaporated to dryness leading to a brown solid with 30–50% yield.[S4]

f) *Synthesis of* CD-a-N-BB

The frozen blackberry seeds and peels provided by Rigoni di Asiago were pre-treated by weighing, rinsing with Milli-Q water for 15 minutes, and air-drying for 24 h. After drying, the residual water was removed by heating in a vacuum-drying chamber (70 °C, 5 mbar, 24 h). In a typical procedure, 2 g of the residue and diethylenetriamine (0.67 g, 6.49 mmol) were put in a Teflon-lined autoclave with 20 mL of MilliQ water and heated at 180 °C for 6 h. The mixture was then filtered (porosity 8–12 μm) and evaporated to dryness leading to a brown and dense oil which was used without further purification.

The obtained compounds were characterized via UV–vis (Figure S1) and TEM (Figure S2).

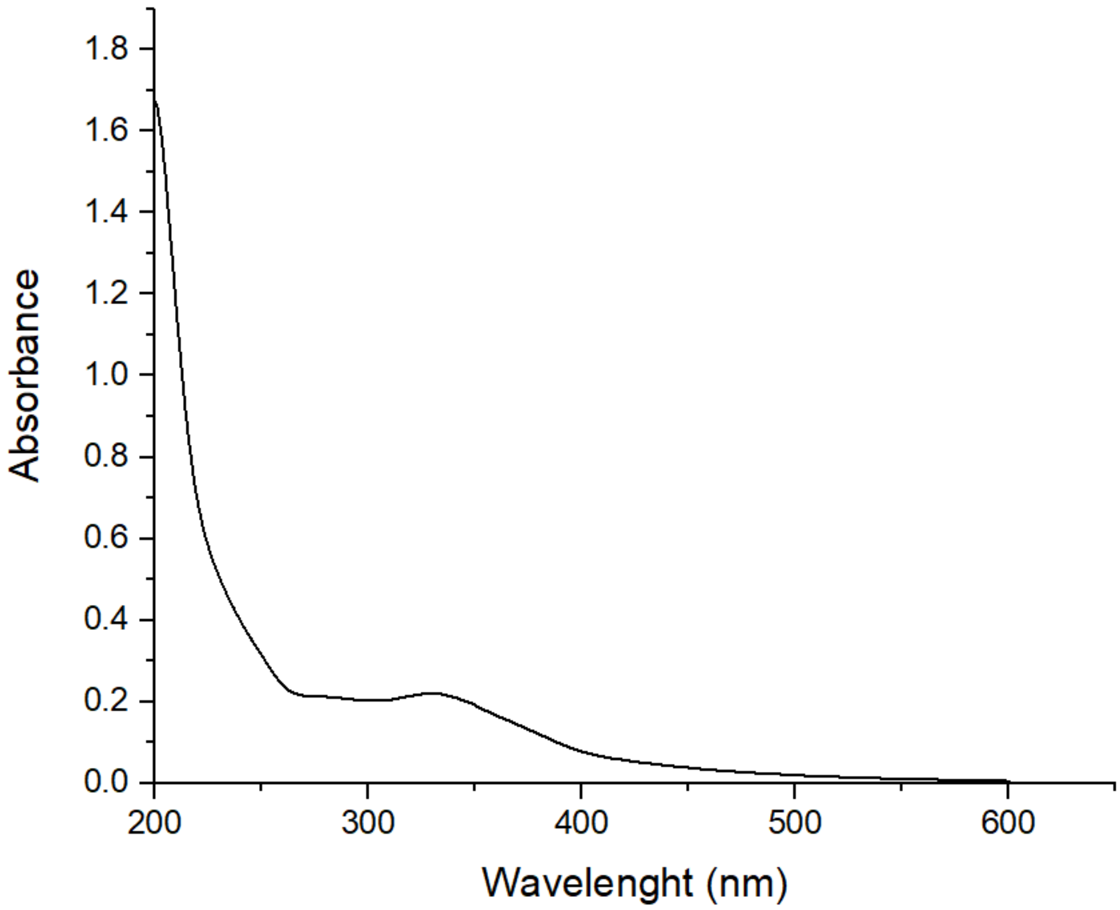

Figure S1: UV–vis absorption spectrum of CDs from blackberries residues (CD-a-N-BB) in neat water.

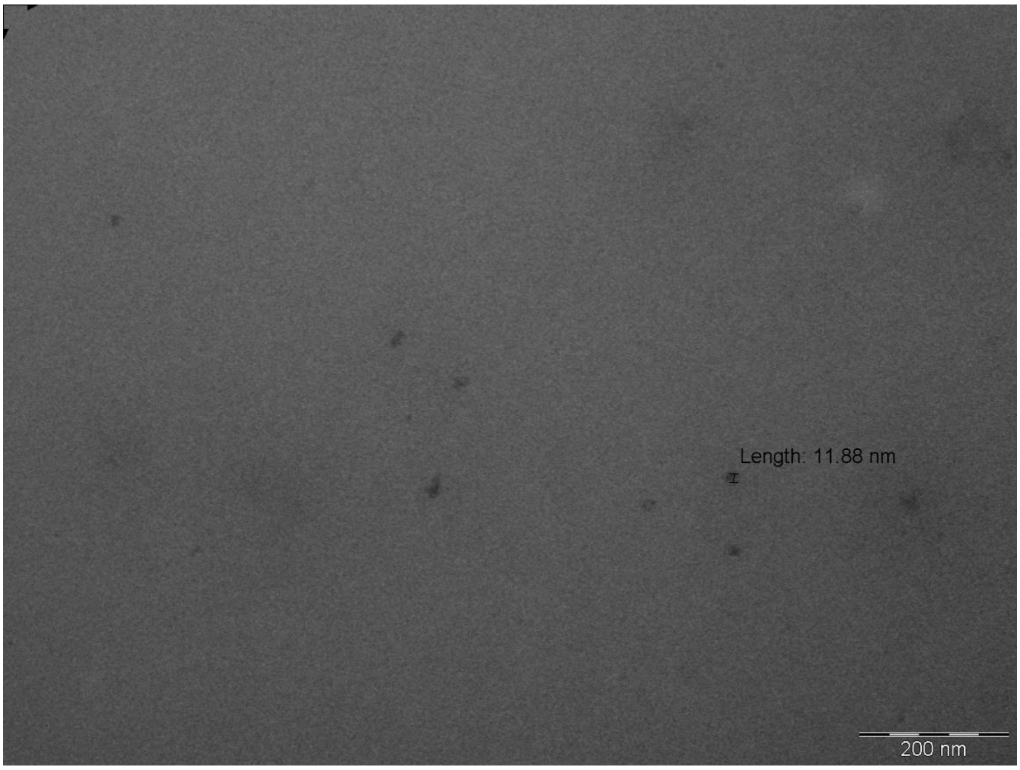

Figure S2: TEM image of carbon dots obtained from *blackberries* residue (CD-a-N-BB).

1.3 Synthesis of triarylsulfonium salts 1.

Compounds **1a**,**b** were commercially available, while compounds **1c** and **1d** were prepared by adapting a method reported in the literature.[S5]

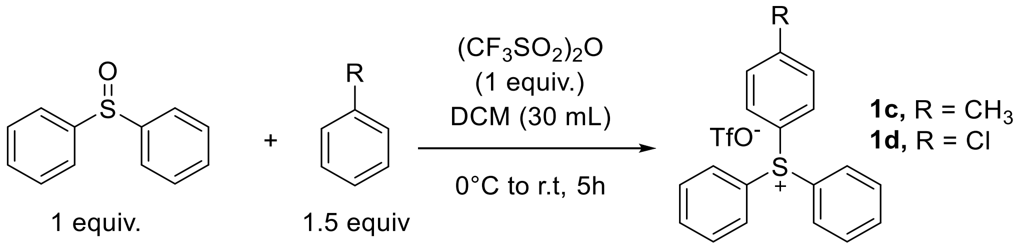

Scheme S1: Synthesis of triaryl sulfonium salts **1c**,**d.**

(4-Methylphenyl)diphenylsulfonium triflate (1c). To a stirred mixture of diphenyl sulfoxide (1.56 g, 7.4 mmol) and toluene (1.18 mL, 1.5 equiv) in DCM (30 mL) was slowly added trifluoromethanesulfonic anhydride (1.24 mL, 1 equiv) at 0 °C. The mixture reacted at room temperature for 5 hours, poured into distilled water (20 mL), and extracted with DCM (3 × 20 mL). The organic layers were evaporated in vacuo and the crude residue purified by silica gel column chromatography (eluent: dichloromethane/acetonitrile mixture 4:1) to provide compound **1c** as yellow solid (1.03 g, 33% yield). The NMR data are in accordance with literature.[S6]

^{1}H NMR (300 MHz, Acetone) δ 7.96 - 7.81 (m, 12H), 7.68-7.65 (m, 2H), 2.50 (s, 3H). ^{13}C NMR (75 MHz, Acetone) δ 147.2, 135.4, 133.1, 132.4, 132.3, 132.0, 126.3, 122.2, 21.5.

4-Chlorophenyldiphenylsulfonium triflate (1d). To a stirred mixture of diphenyl sulfoxide (1.56 g, 7.4 mmol) and chlorobenzene (1.14 mL, 1.5 equiv) in DCM (30 mL) was slowly added trifluoromethanesulfonic anhydride (1.24 mL, 1 equiv) at 0 °C. The mixture reacted at room temperature for 5 hours, poured into distilled water (20 mL), and extracted with DCM (3 × 20 mL). The organic layers have been evaporated in vacuo and the crude residue purified by silica gel column chromatography (eluent: dichloromethane/acetonitrile mixture 4:1) to provide compound **1d** as white solid m.p. 95.5 °C (0.333 g, 10% yield). Compound was not stable for the HRMS analysis. ^{1}H NMR (300 MHz, acetone) δ 8.04–7.79 (m, 14H). ^{13}C NMR (75 MHz, Acetone) δ 141.7, 135.7, 134.0, 132.6, 132.3, 125.7, 124.7.

1.4 Light-mediated reduction of triarylsulfonium salts 1a–d

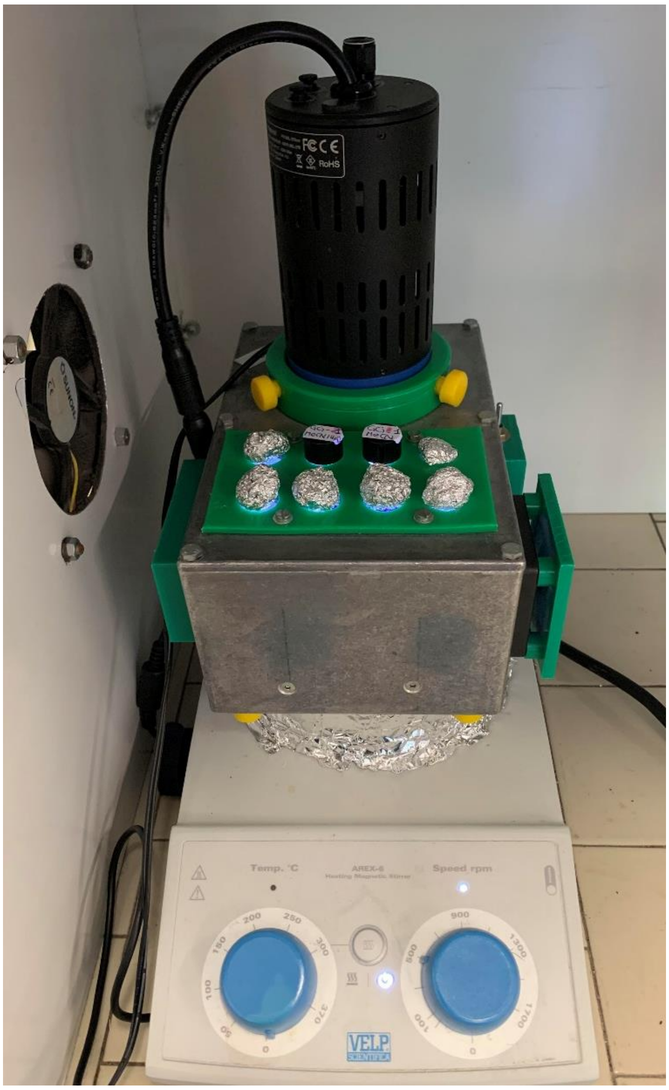

Figure S3: Glass vessel containing the reaction mixture, equipped with a magnetic stirrer and closed with a screw cap in a homemade photoreactor equipped with a lateral fan, on a magnetic plate and irradiated with a 40 W Kessil lamp (full power; emission centered at 427 nm).

Photochemical experiments on compound 1a. In a glass vessel equipped with a magnetic stirrer, to a solution of CD a-N-Cit (20 mg/mL) in THF/water 1:1 (1 mL) was added (41.2 mg, 0.1 mmol, 0.1 M) of compound **1a**. The solution obtained was saturated with N_2 and then irradiated in a homemade photoreactor on magnetic plate, at room temperature using a 40 W Kessil lamp (full power, emission centered at 427 nm) for 24 hours. The crude residue was evaporated under vacuo and the compound purified by silica flash column chromatography, using cyclohexane as eluent, to afford the desired product (**2a**) as colorless oil (7.8 mg, 42% yield). The NMR spectral data are in accordance with the

literature[7]; ^{1}H NMR (300 MHz, DMSO) δ 7.42-7.28 (m, 10H). ^{13}C NMR (75 MHz, DMSO) δ 134.8, 130.7, 129.5, 127.4.

The same procedure was followed by testing the other CDs (CD-a-CIT, CD-a-GLU, CD-g-CIT, CD-a-BASS, CD-a-N-BB) and the yield of biaryls was quantified by analyzing the reaction solution with GC-FID using dodecane as internal standard. The yields are reported in Table S1.

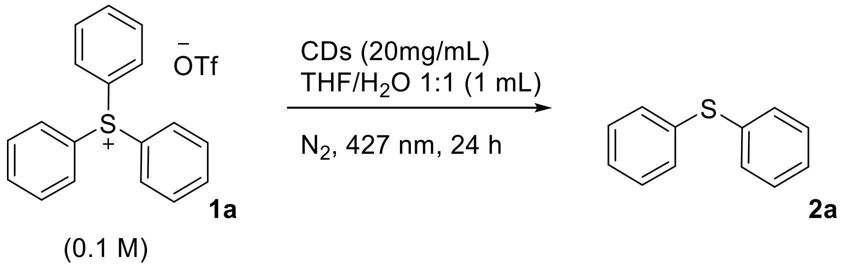

Sulf. Salt		Cdots type	Product	Yield %
**1a**	*R₁ = H*	CD-a-N-CIT	**2a** *R_1 = H, R_2 = H*	42[a]
	R_2 = H	CD-a-CIT	**2a** *R_1 = H, R_2 = H*	54[b]
		CD-g-CIT	**2a** *R_1 = H, R_2 = H*	7[b]
		CD-a-GLU	**2a** *R_1 = H, R_2 = H*	55[b]
		CD-a-BASS	**2a** *R_1 = H, R_2 = H*	7[b]
		CD-a-N-BB	**2a** *R_1 = H, R_2 = H*	10[b]

Table S1: Comparison of the yields of the photoreduction's product of compound **1a** with the different CDs. [a.] Isolated yields [b.] GC-yields using dodecane as internal standard

Photochemical experiments on compound 1b. In a glass vessel equipped with a magnetic stirrer, to a solution of CD-a-N-Cit (20 mg/mL) in THF/water 1:1 (1 mL) was added (45.4 mg, 0.1 mmol, 0.1 M) of compound **1b**. The obtained solution was degassed with N_2 and then irradiated in a homemade photoreactor on magnetic plate, at room temperature using a 40 W Kessil lamp (full power, emission centered at 427 nm) for 24 hours. The crude residue was evaporated under vacuo and the compound purified by silica flash column chromatography, using cyclohexane as eluent, to afford the desired product (**2b**) white solid m.p. 54°C (14.7 mg, 69% of yield). The NMR data are in accordance with literature[8] ^{1}H NMR (300 MHz, DMSO) δ 7.27-7.10 (m, 8H), 2.28 (s, 6H). ^{13}C NMR (75 MHz, DMSO) δ 136.8, 131.7, 130.7, 130.1, 20.6.

The same procedure was followed by testing the other CDs (CD-a-CIT, CD-a-GLU, CD-g-CIT, CD-a-BASS, CD-a-N-BB) and the yield of biaryls was quantified by analyzing the reaction solution with GC-FID using dodecane as internal standard. The results have been reported in Table S2.

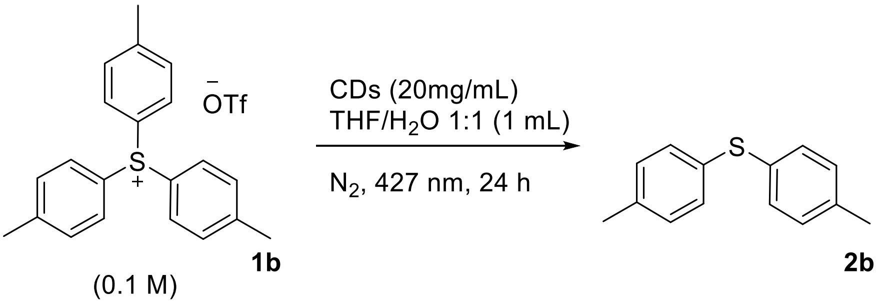

Compound		CDs	Product	Yield %
**1b**	$R_1 = CH_3$	CD-a-N-CIT	**2b** $R_1 = CH_3, R_2 = CH_3$	69[a]
	$R_2 = CH_3$	CD-a-CIT	**2b** $R_1 = CH_3, R_2 = CH_3$	61[b]
		CD-g-CIT	**2b** $R_1 = CH_3, R_2 = CH_3$	9[b]
		CD-a-GLU	**2b** $R_1 = CH_3, R_2 = CH_3$	33[b]
		CD-a-BASS	**2b** $R_1 = CH_3, R_2 = CH_3$	3[b]
		CD-a-N-BB	**2b** $R_1 = CH_3, R_2 = CH_3$	11[b]

Table S2: Comparison of the yields of the photoreduction's product of compound **1b** with the different CDs. [a]Isolated yields [b]GC-yields using dodecane as internal standard

Photochemical experiments on compound 1c. In a glass vessel equipped with a magnetic stirrer, to a solution of CD-a-CIT (20 mg/mL) in THF/water 1:1 (3 mL) were added (127.8 mg, 0.3 mmol, 0.1 M) of compound **1c**. The obtained solution was degassed with N_2 and then irradiated in a homemade photoreactor on magnetic plate, at room temperature using a 40 W Kessil lamp (full power, emission centered at 427 nm) for 24 hours. The crude residue was evaporated under vacuo and the mix of the two products **2a** and **2c** purified by silica flash column chromatography, using cyclohexane as eluent, to afford to afford a mixture (27.2 mg) of the products **2a** (7.6 mg, 14% of yield) and **2c** (19.6 mg, 33% of yield).

^{1}H NMR (300 MHz, $DMSO_3$) δ 7.40-7.17 (m, 13H), 2.30 (s, 3H). ^{13}C NMR (75 MHz, DMSO) δ 137.6, 136.1, 134.8, 132.0, 130.7, 130.3, 129.6, 129.4, 129.4, 127.4, 126.7, 21.2.

The same procedure was followed by testing the other CDs (a-CIT, a-GLU, g-CIT, BASS, BB and the yield of biaryls was quantified by analyzing the reaction solution with GC-FID using dodecane as internal standard. The yields are reported in Table S3.

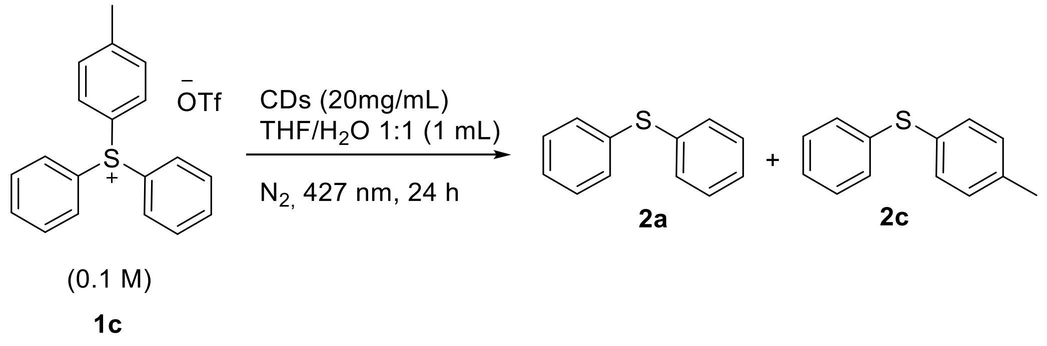

Compound		CDs	Product	Yield %
**1c**	$R_1 = H$	CD-a-N-CIT	**2a** $R_1 = H, R_2 = H$	9[b]
	$R_2 = CH_3$		**2c** $R_1 = CH_3, R_2 = H$	37[b]
		CD-a-CIT	**2a** $R_1 = H, R_2 = H$	14[a]
			**2c** $R_1 = CH_3, R_2 = H$	33[a]
		CD-g-CIT	**2a** $R_1 = H, R_2 = H$	3[b]
			**2c** $R_1 = CH_3, R_2 = H$	4[b]
		CD-a-GLU	**2a** $R_1 = H, R_2 = H$	14[b]
			**2c** $R_1 = CH_3, R_2 = H$	31[b]
		CD-a-BASS	**2a** $R_1 = H, R_2 = H$	4[b]
			**2c** $R_1 = CH_3, R_2 = H$	6[b]
		CD-a-N-BB	**2a** $R_1 = H, R_2 = H$	7[b]
			**2c** $R_1 = CH_3, R_2 = H$	10[b]

Table S3: Comparison of the yields of the photoreduction's product of compound **1c** with the different CDs. a. Isolated yields b. GC-yields using dodecane as internal standard.

Photochemical experiments on compound 1d. In a glass vessel equipped with a magnetic stirrer, to a solution of CD-a-N-CIT (20 mg/mL) in THF / water 1:1 (2 mL) were added (89.6 mg, 0.2 mmol, 0.1 M) of compound **1d**. The obtained solution was degassed with N_2 and then irradiated in a homemade photoreactor on magnetic plate, at room temperature using a 40W Kessil lamp (full power, emission centered at 427 nm) for 24 hours. The crude residue was evaporated under vacuo and the mix of the two products **2a** and **2d** purified by silica flash column chromatography, using cyclohexane as eluent, to afford a mixture 32.1 mg of **2a** (13.2 mg, 36% of yield) and **2d** (18.9 mg, 43% of yield).

^{1}H NMR (300 MHz, DMSO) δ 7.44-7.28 (m, 17.3H). ^{13}C NMR (75 MHz, DMSO) δ 134.8, 134.3, 133.9, 132.0, 131.9, 131.3, 130.7, 129.7, 129.5, 129.5, 127.9, 127.4.

The same procedure was followed by testing the other CDs (CD-a-CIT, CD-a-GLU, CD-g-CIT, CD-a-BASS, CD-a-N-BB) and the yield of biaryls was quantified by analyzing the reaction solution with GC-FID using dodecane as internal standard. The yields are reported in Table S4.

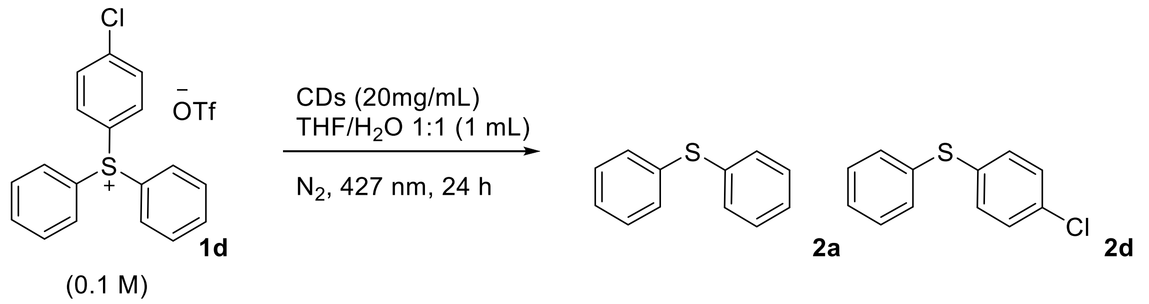

Compound		CDs	Product	Yield %
**1d**	*R₁ = H*	CD-a-N-CIT	**2a** *R_1 = H, R_2 = H*	36[a]
	R_2 = Cl		**2d** *R_1 = Cl, R_2 = H*	43[a]
		CD-a-CIT	**2a** *R_1 = H, R_2 = H*	27[b]
			**2d** *R_1 = Cl, R_2 = H*	30[b]
		CD-g-CIT	**2a** *R_1 = H, R_2 = H*	5[b]
			**2d** *R_1 = Cl, R_2 = H*	5[b]
		CD-a-GLU	**2a** *R_1 = H, R_2 = H*	33[b]
			**2d** *R_1 = Cl, R_2 = H*	33[b]
		CD-a-BASS	**2a** *R_1 = H, R_2 = H*	11[b]
			**2d** *R_1 = Cl, R_2 = H*	12[b]
		CD-a-N-BB	**2a** *R_1 = H, R_2 = H*	12[b]
			**2d** *R_1 = Cl, R_2 = H*	12[b]

Table S4: Comparison of the yields of the photoreduction's product of compound **1d** with the different CDs. a. Isolated yields b. GC-yields using dodecane as internal standard.

1.5 Photophysics of CDs

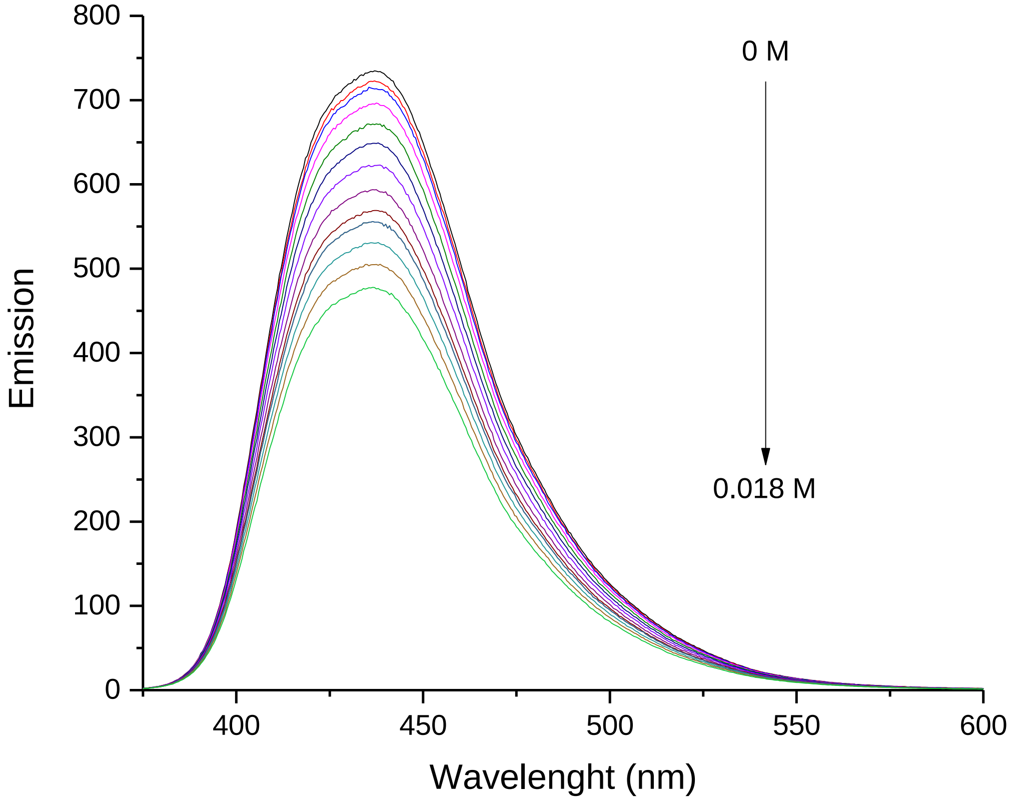

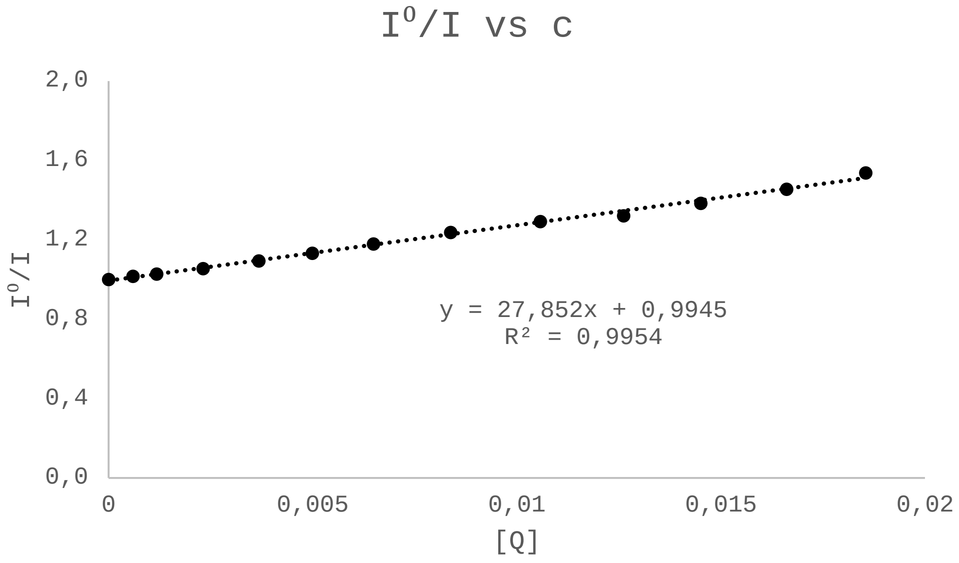

Figure S4: Interaction of a-N-Cit in THF/H_2O 1:1 mixture (λ_{ex} = 354 nm, Abs = 0.197) with **1b** $K_q(I) = 2.14 \times 10^{10}\ M^{-1}\ s^{-1}$.

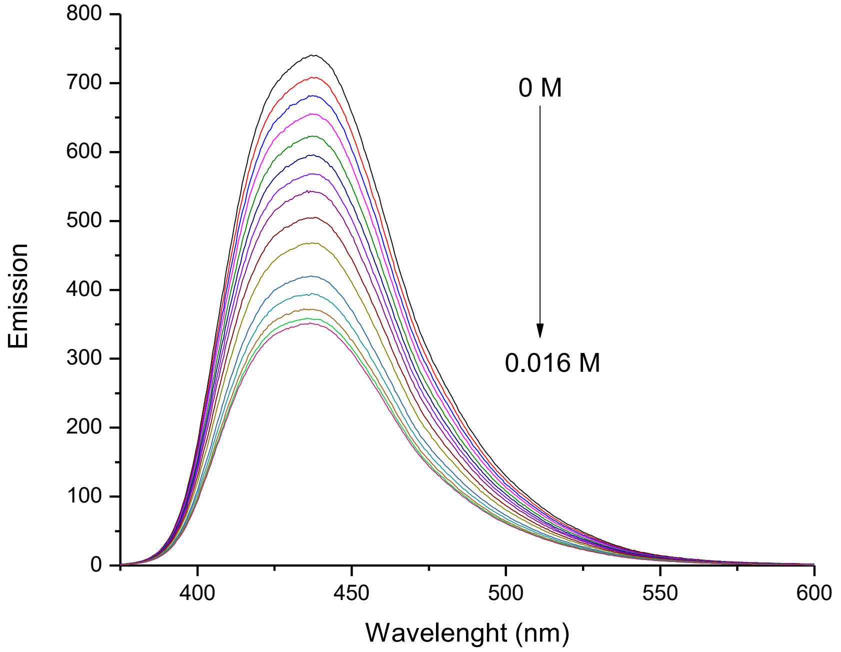

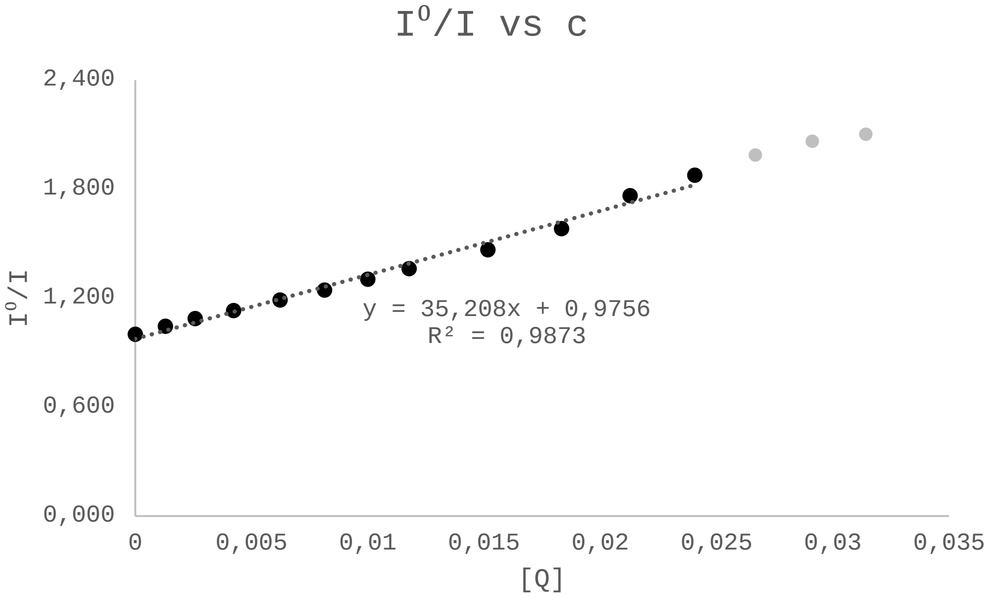

Figure S5: Interaction of a-Cit in THF/H_2O 1:1 mixture (λ_{ex} = 358 nm, Abs = 0.206) with **1b**. $K_q(I) = 9.78 \times 10^{10} M^{-1} s^{-1}$.

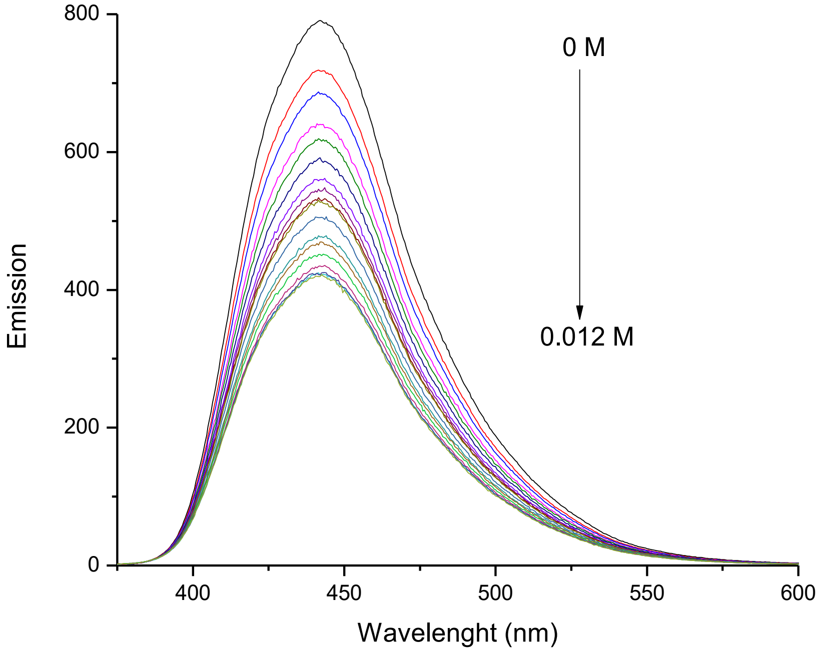

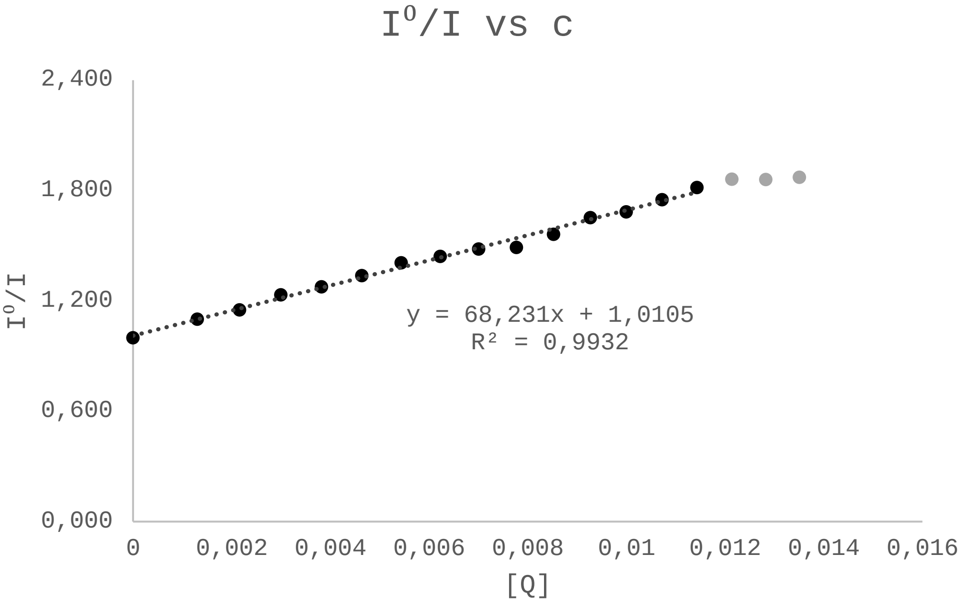

Figure S6: Quench of a g-Cit solution in THF/H_2O 1:1 mixture (λ_{ex} = 353 nm, Abs = 0.163) with a **1b** solution. $K_q(I) = 1.6 \times 10^{11}\ M^{-1}\ s^{-1}$**.**

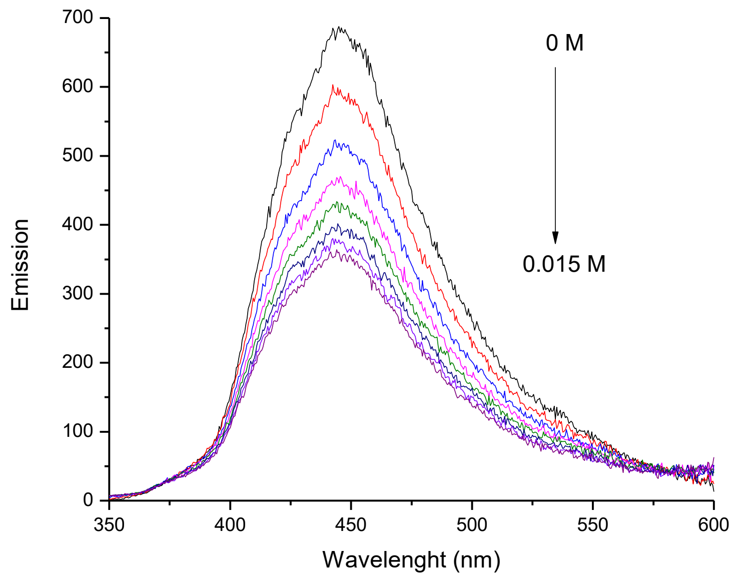

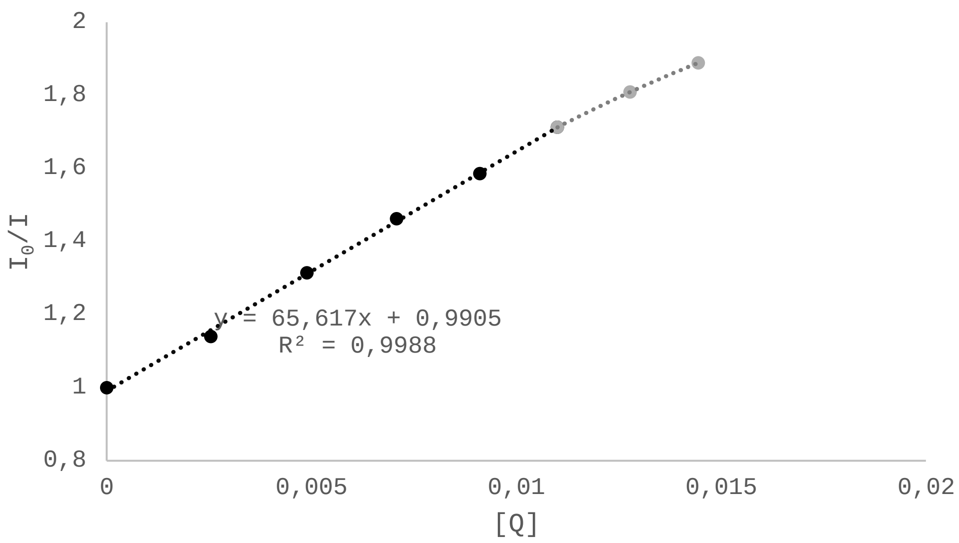

Figure S7: Quench of a a-N-BB solution in THF/H_2O 1:1 mixture (λ_{ex} = 330 nm, Abs = 0.220) with a **1b** solution. $K_q(I) = 1.7 \times 10^{10}\ M^{-1}\ s^{-1}$.

Time-resolved photoluminescence (PL) measurements

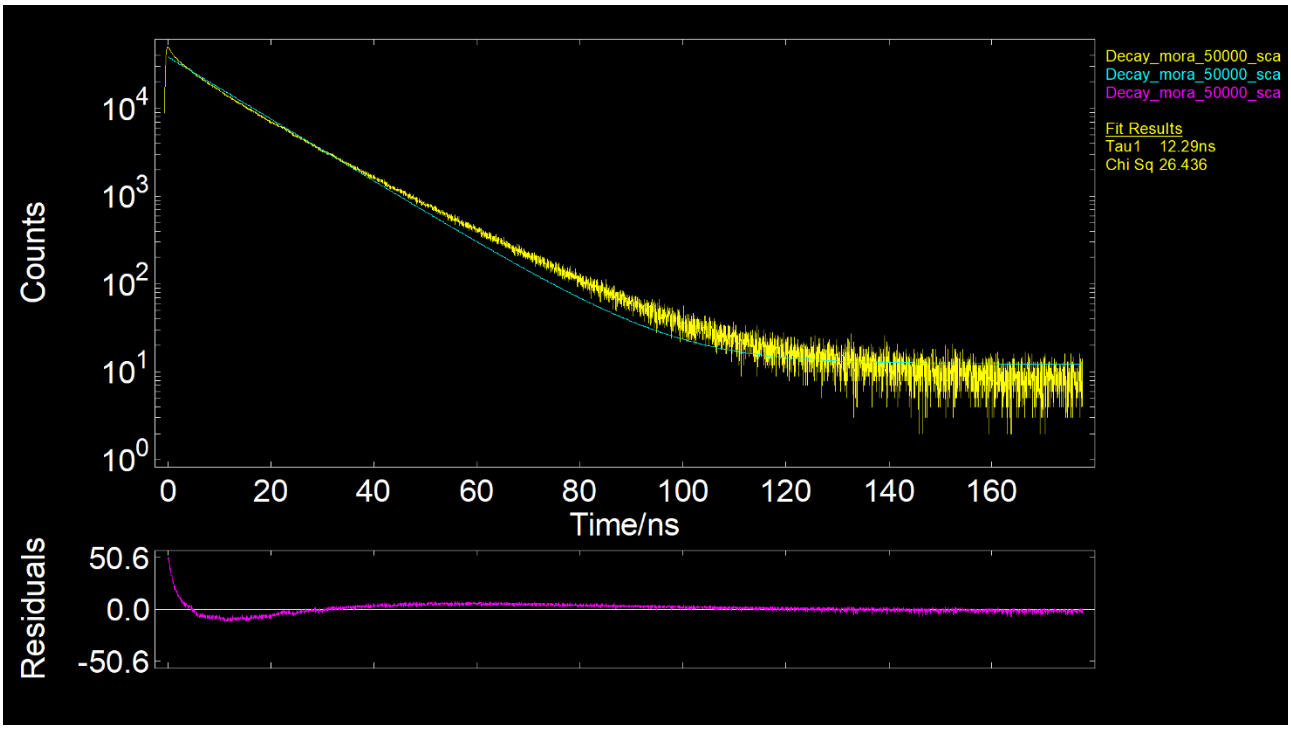

Figure S8: Time-resolved PL spectra of a-N-BB analysed with a mono exponential fitting

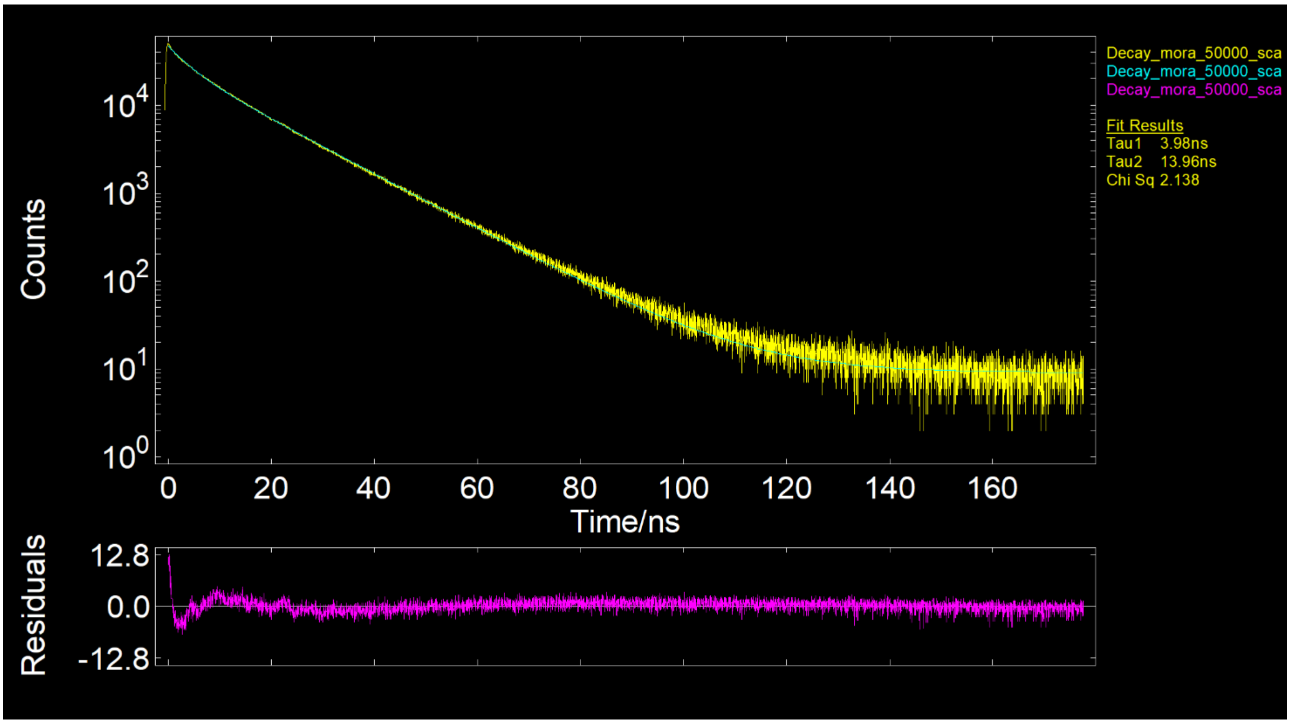

Figure S9: Time-resolved PL spectra of CD-a-N-BB analyzed with a biexponential fitting

1.6 Cyclic voltammetry of triarylsulfonium salts.

Cyclic voltammetry of triarylsulfonium salts was carried out by means of a Amel model 4330 module equipped with a 5 mL standard three-electrode cell with a glassy carbon (0.49 cm^2 geometrical area) working electrode, a platinum wire as auxiliary electrode and an Ag/AgCl, 3 M NaCl reference electrode, all obtained from BASi Electrochemistry. A solution of DMF and 200 µL of water containing 0.1 M lithium perchlorate was used as solvent and supporting electrolyte, scanning the potential in the range from 0 to −2400 mV, with a 5 mM compound concentration and a scan speed of 100 mVs^{-1}.

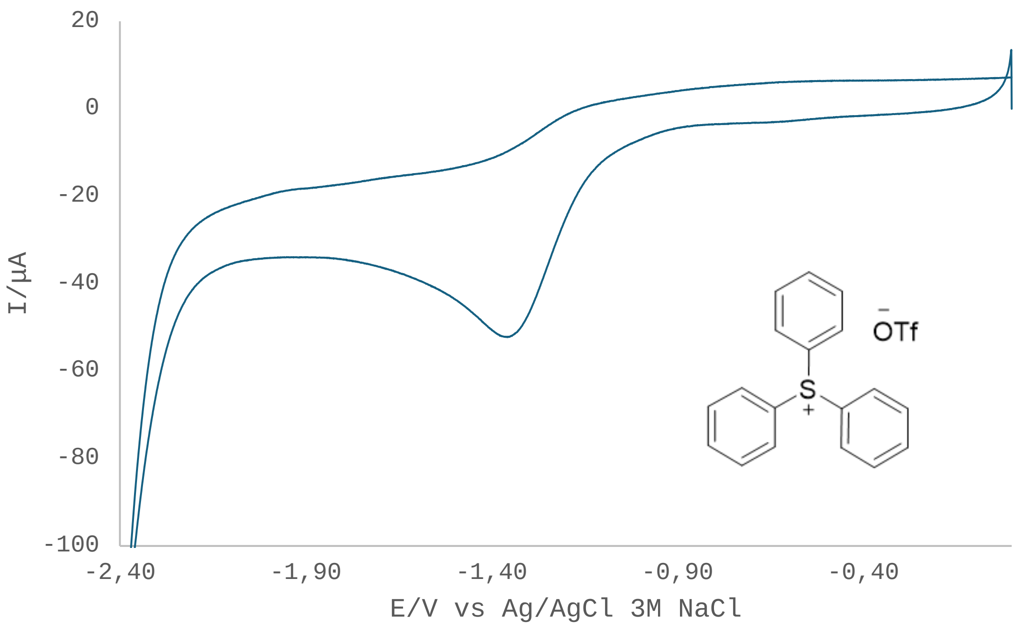

E°(V) = -1.35 vs Ag/AgCl

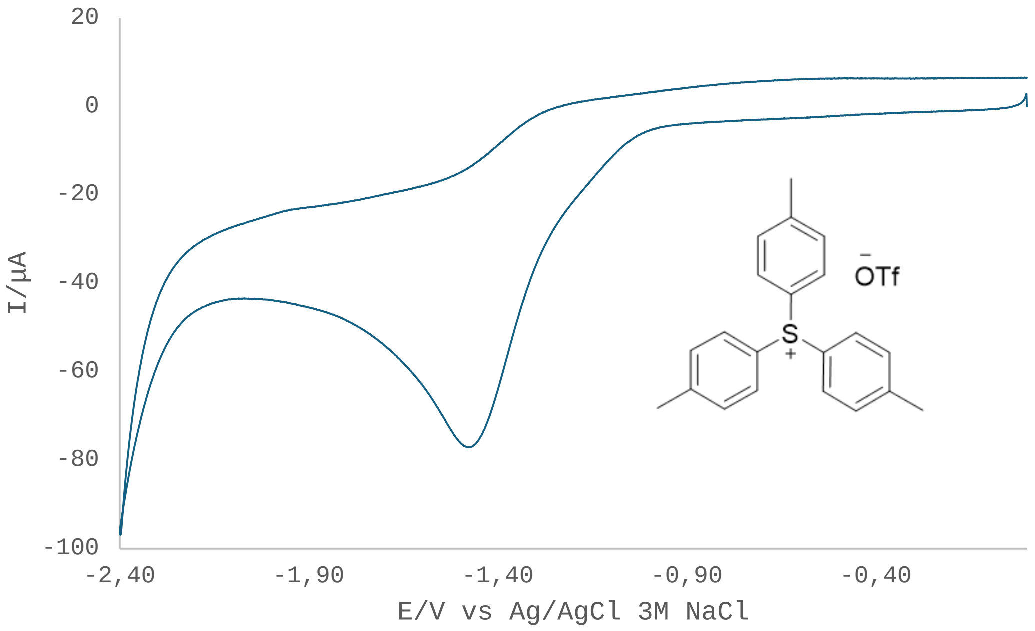

E°(V) = -1.44 vs Ag/AgCl

Figure S10: Cyclic voltammetry of a DMF solution of **1a** (upper figure) and **1b** (down)

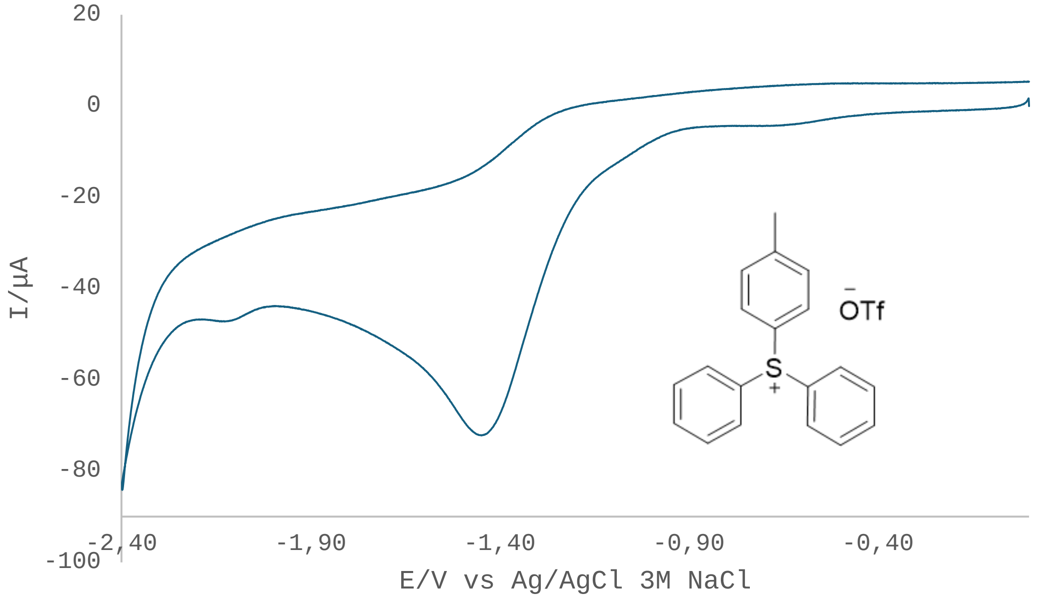

E°(V) = -1.46 vs Ag/AgCl

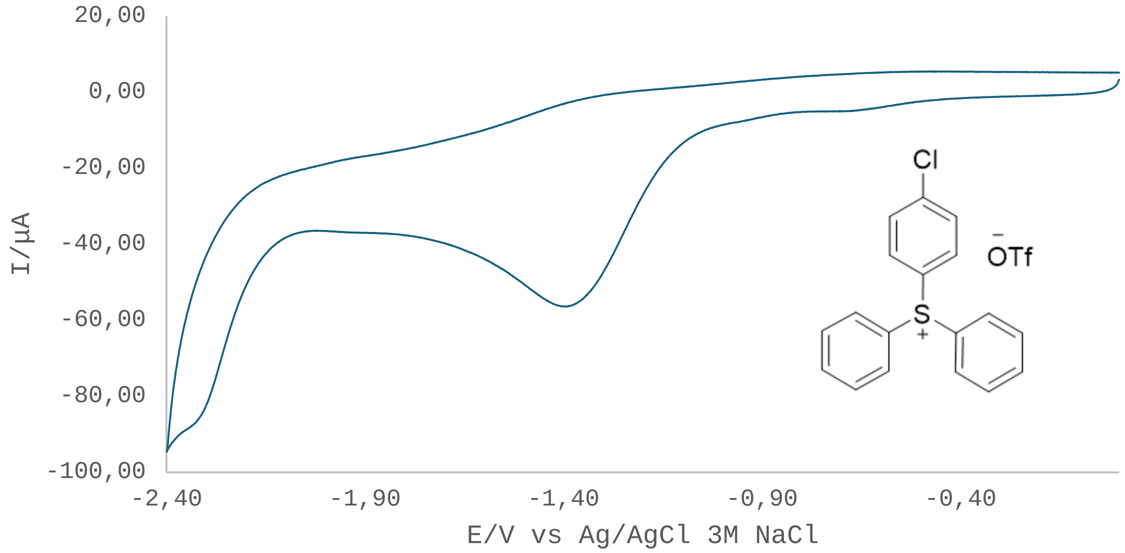

E°(V) = -1.39 vs Ag/AgCl

Figure S10: Cyclic voltammetry of a DMF solution of **1c** (upper figure) and **1c** (down)

2. References

S1 S. Cailotto, R. Mazzaro, F. Enrichi, A. Vomiero, M. Selva, E. Cattaruzza, D. Cristofori, E. Amadio, A. Perosa, Design of Carbon Dots for Metal-free Photoredox Catalysis, *ACS Appl. Mater. Interfaces* **2018**, *10*, 40560–40567.

S2 S. Cailotto, M. Negrato, S. Daniele, R. Luque, M. Selva, E. Amadio, A. Perosa, Carbon dots as photocatalysts for organic synthesis: metal-free methylene–oxygen-bond photocleavage, *Green Chem.* **2020**, *22*, 1145–1149.

S3 S. Cailotto, E. Amadio, M. Facchin, M. Selva, E. Pontoglio, F. Rizzolio, P. Riello, G. Toffoli, A. Benedetti, A. Perosa, Fluorescent Carbon Nanoparticles in Medicine for Cancer Therapy: An Update, *ACS Med. Chem. Lett.* **2018**, *9*, 832–837.

S4 C. Campalani, E. Cattaruzza, S. Zorzi, A. Vomiero, S. You, L. Matthews, M. Capron, C. Mondelli, M. Selva, A. Perosa, Biobased Carbon Dots: From Fish Scales to Photocatalysis, *Nanomaterials* **2021**, *11*, 524.

S5 Z.-Y. Tian, S.-M. Wang, S.-J. Jia, H.-X. Song, C.-P. Zhang, Sonogashira Reaction Using Arylsulfonium Salts as Cross-Coupling Partners, *Org. Lett.* **2017**, *19*, 5454–5457.

S6 N. Takenaga, Y. Yoto, T. Hayashi, N. Miyamoto, H. Nojiri, R. Kumar, T. Dohi, Part (vii): Special Issue 'Hypervalent Iodine Chemistry, part 3, *ARKIVOC* **2022**, *2022*, 7–18.

S7 X. Gao, F. Chen, M. Y. Jin, C. Xu, Triethyl amine as an effective reducing agent for sulfoxide deoxygenation, *Org. Biomol. Chem.* **2024**, *22*, 3215–3219.

S8 J.-X. Li, R. Tian, Y.-M. Zhu, Asymmetric autocatalysis with amplification of enantiomeric excess utilizing chiral crystals of achiral o-terphenyls as chiral triggers, *Tetrahedron* **2024**, *152*, 133814

3. Copies of ^{1}H and ^{13}C spectra of the compounds synthesized in the present work.

(4-Methylphenyl)diphenylsulfonium triflate (1c)

^{1}H NMR (300 MHz)

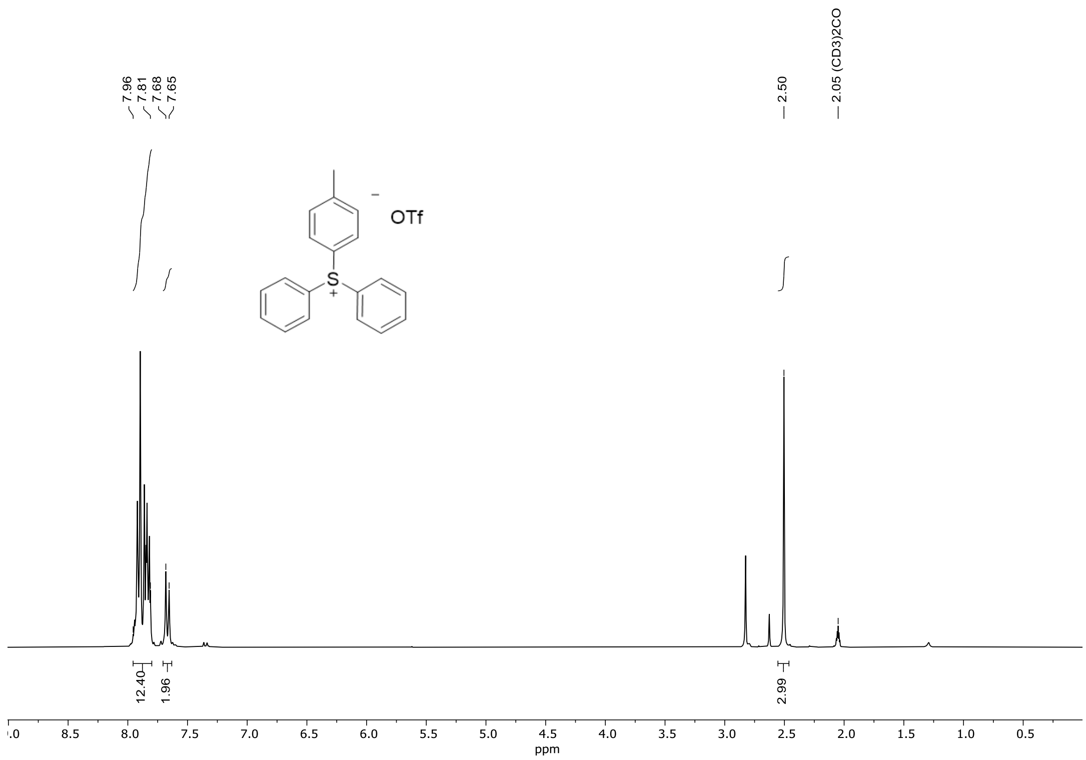

^{13}C NMR (75 MHz)

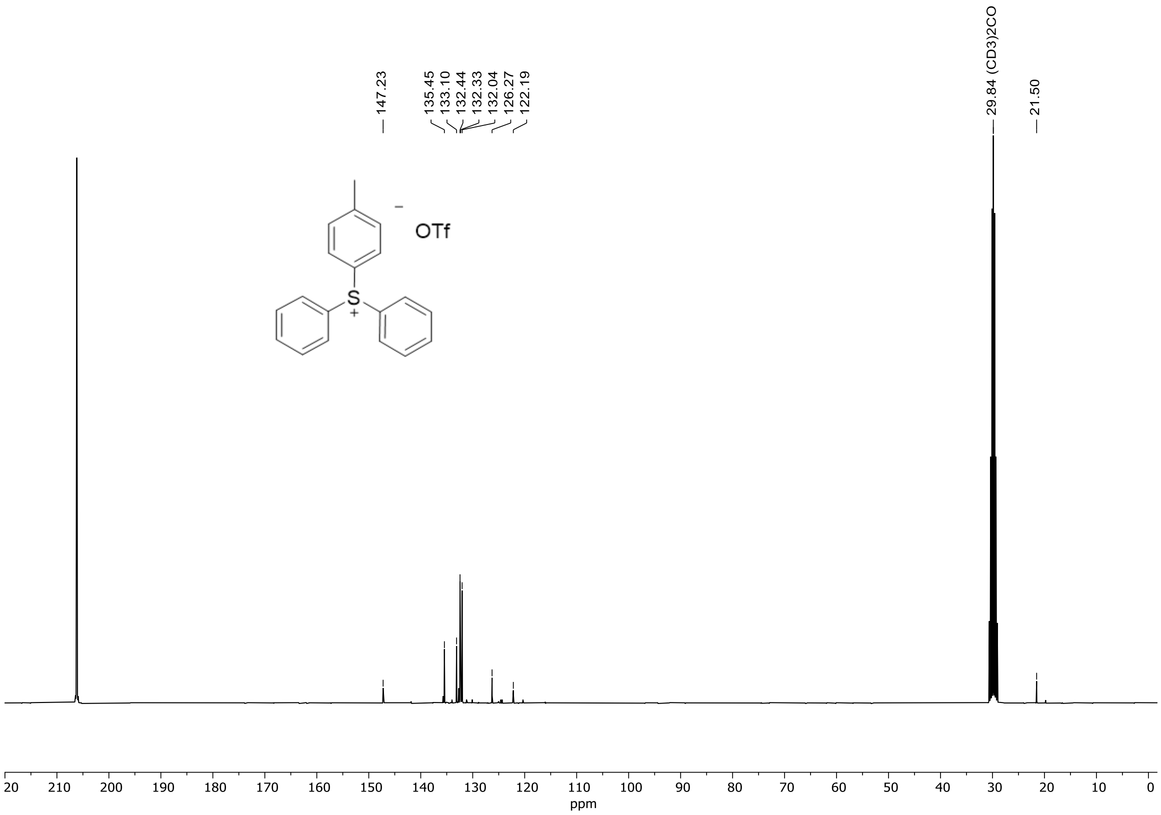

4-Chlorophenyldiphenylsulfonium triflate (1d)

^{1}H NMR (300 MHz)

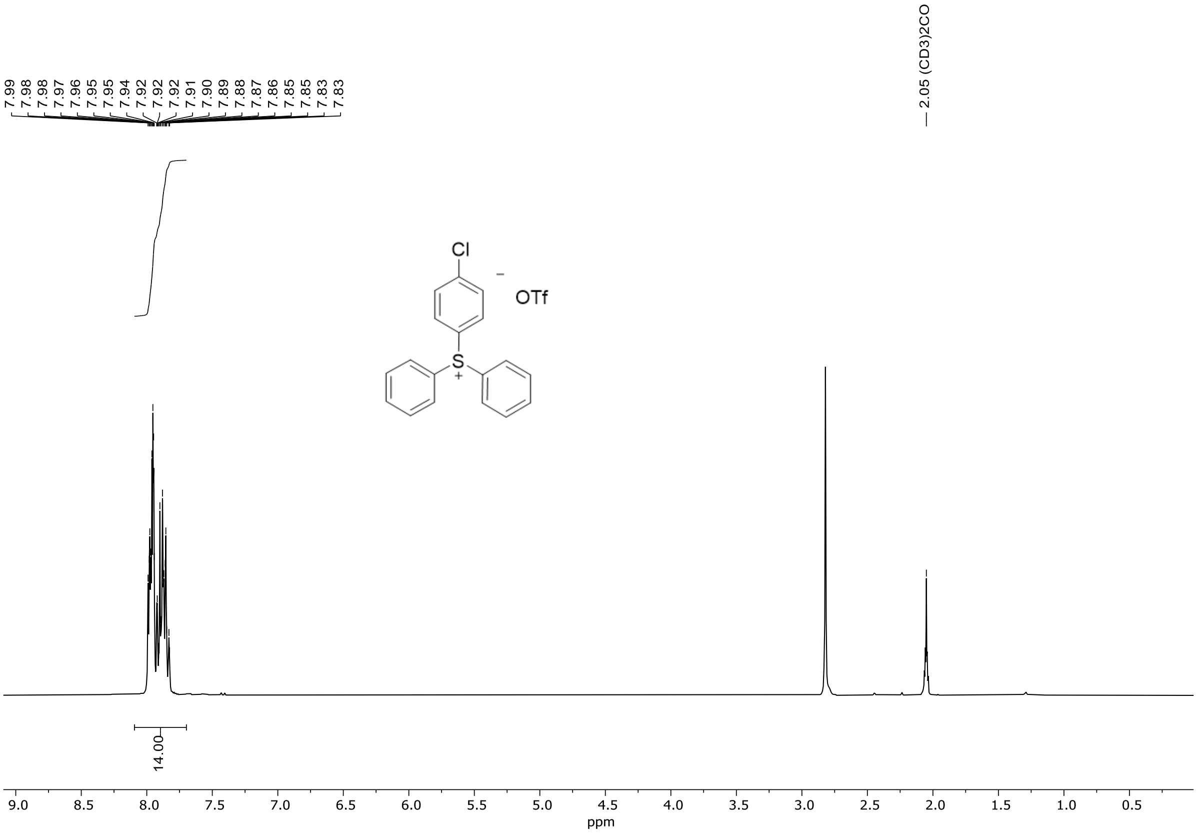

^{13}C NMR (75 MHz)

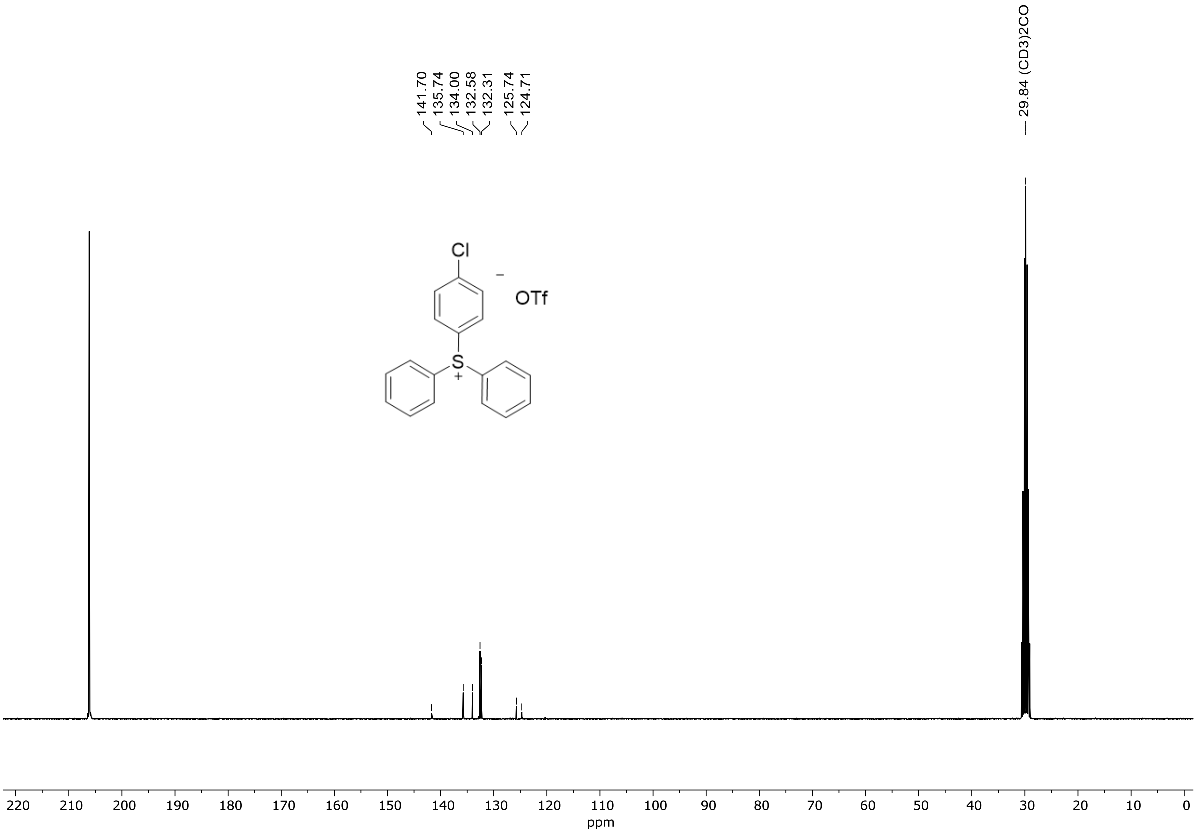

Diphenyl sulfide (2a)

^{1}H NMR (300 MHz)

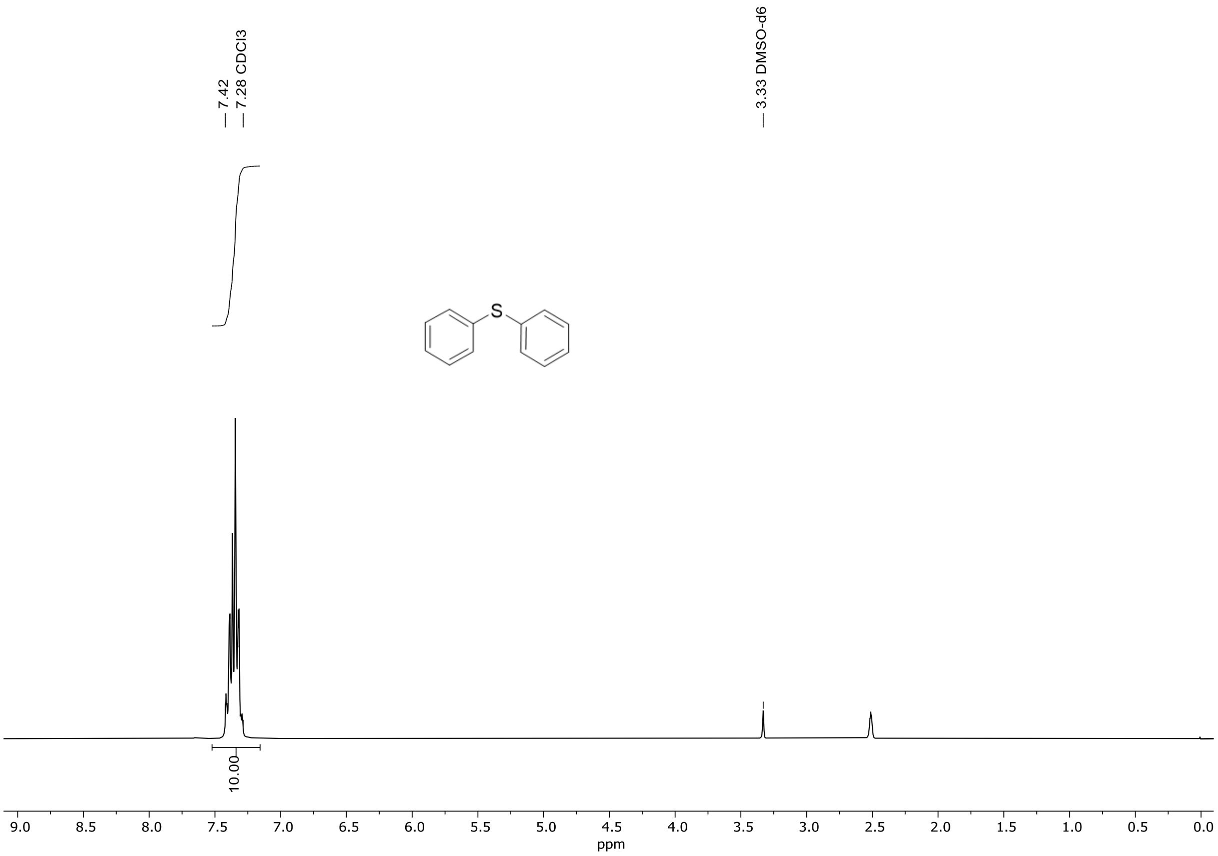

^{13}C NMR (75 MHz)

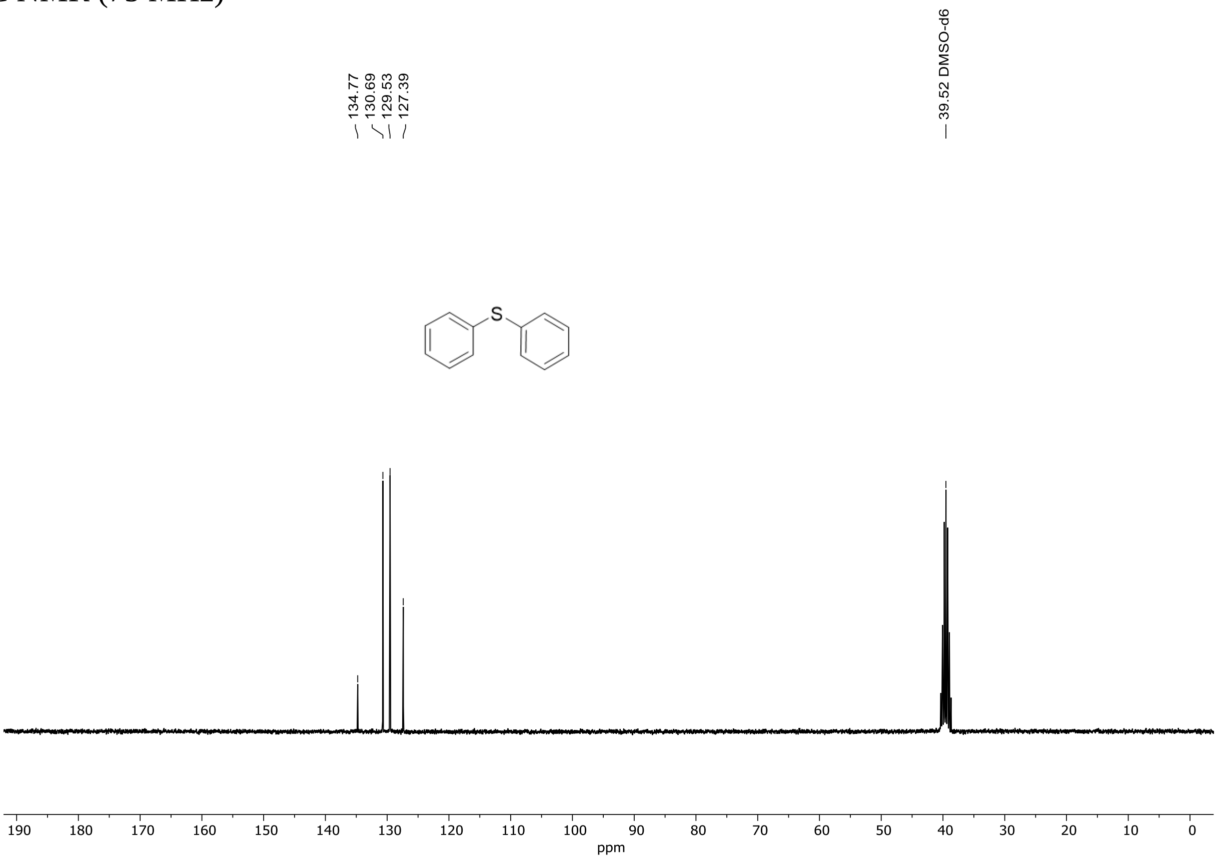

Di-*p*-tolylsulfane (2b)

^{1}H NMR (300 MHz)

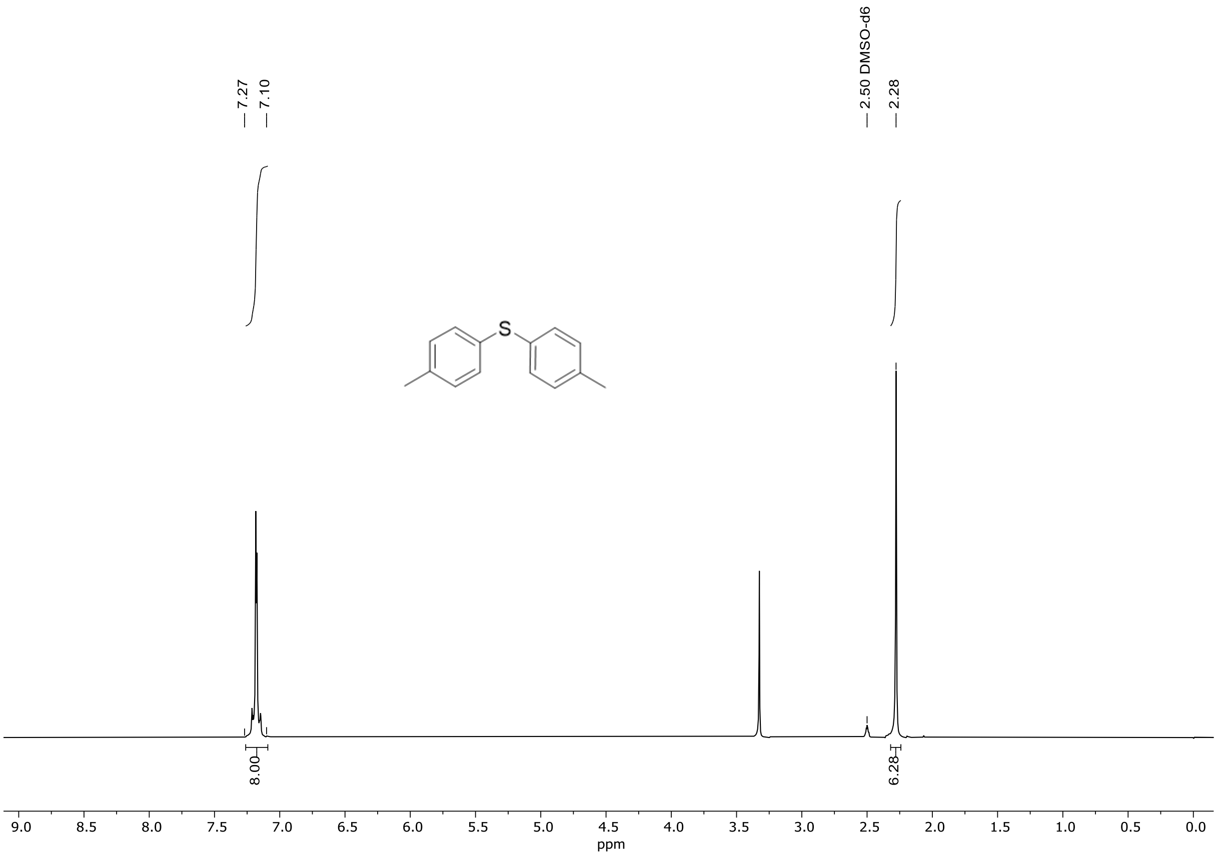

^{13}C NMR (75 MHz)

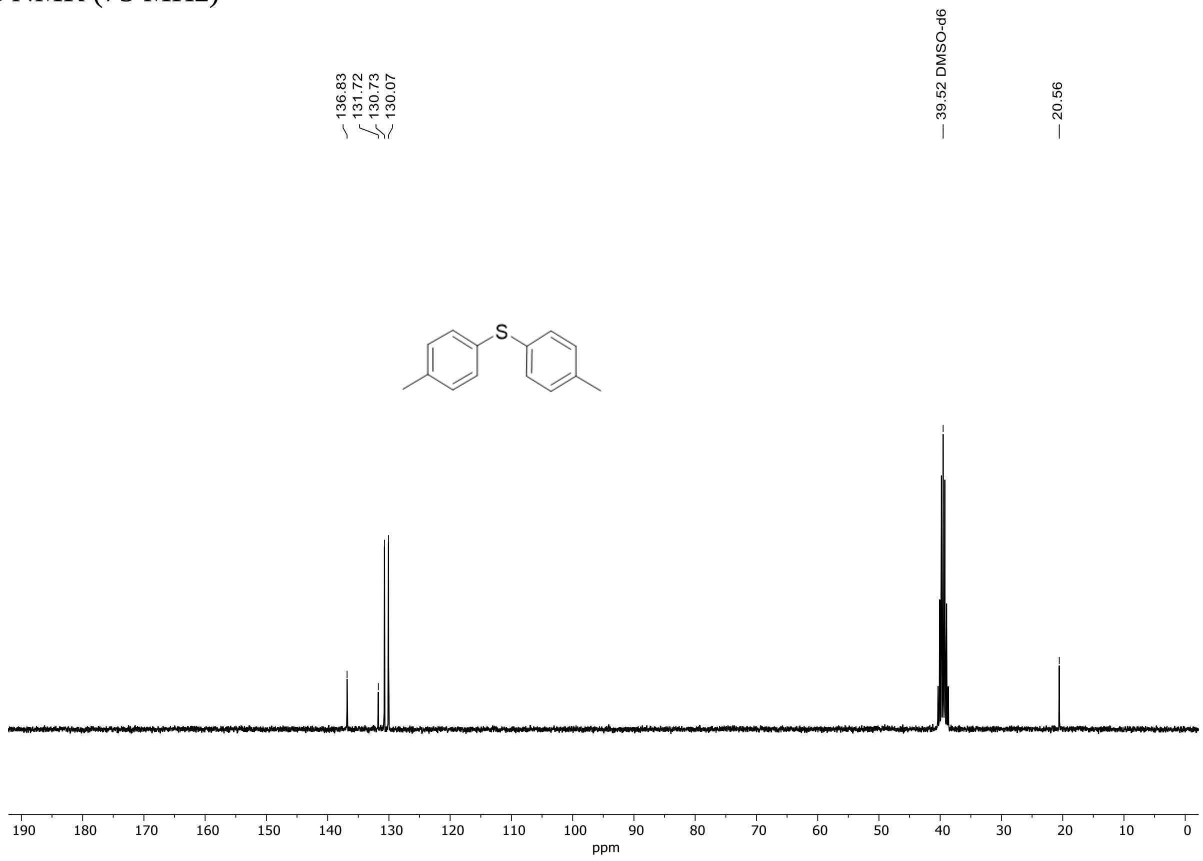

Diphenyl sulfide (2a), phenyl(p-tolyl)sulfane (2c)

^{1}H NMR (300 MHz)

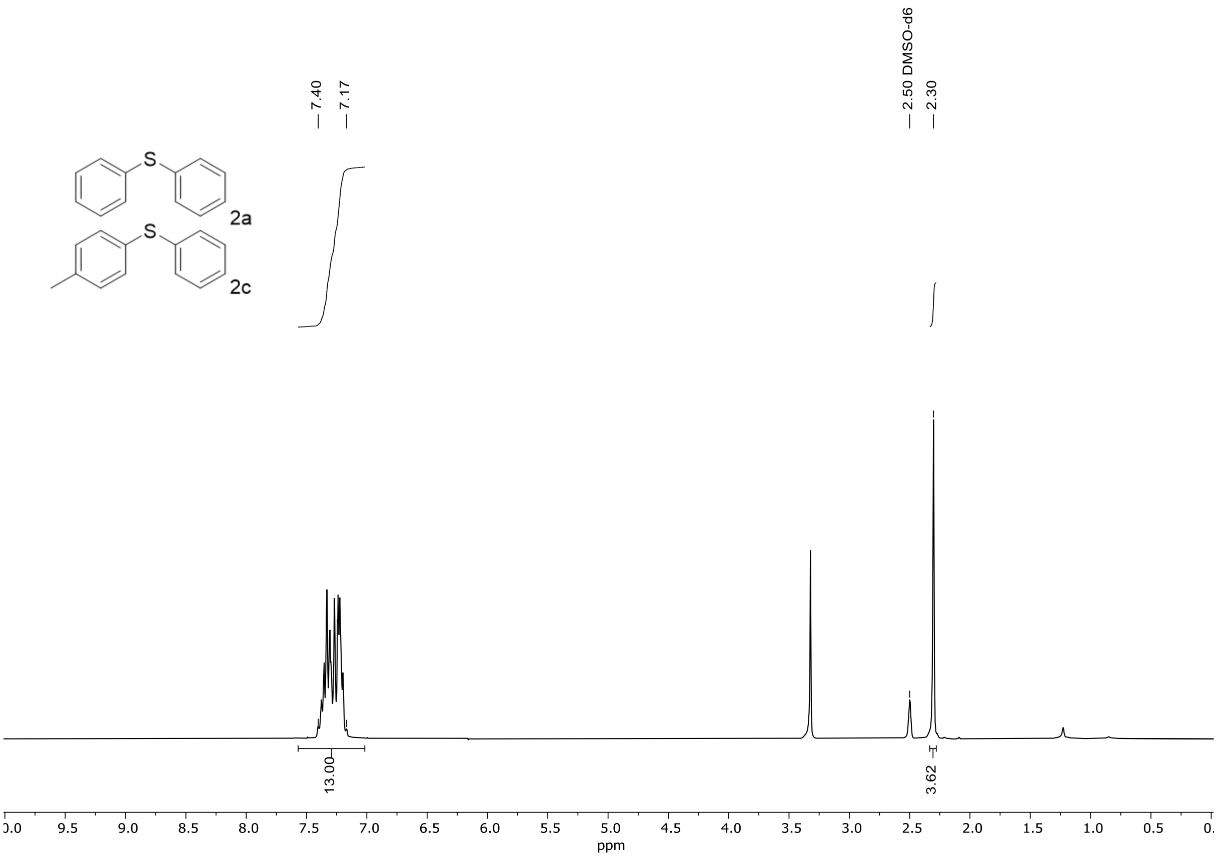

^{13}C NMR (75 MHz)

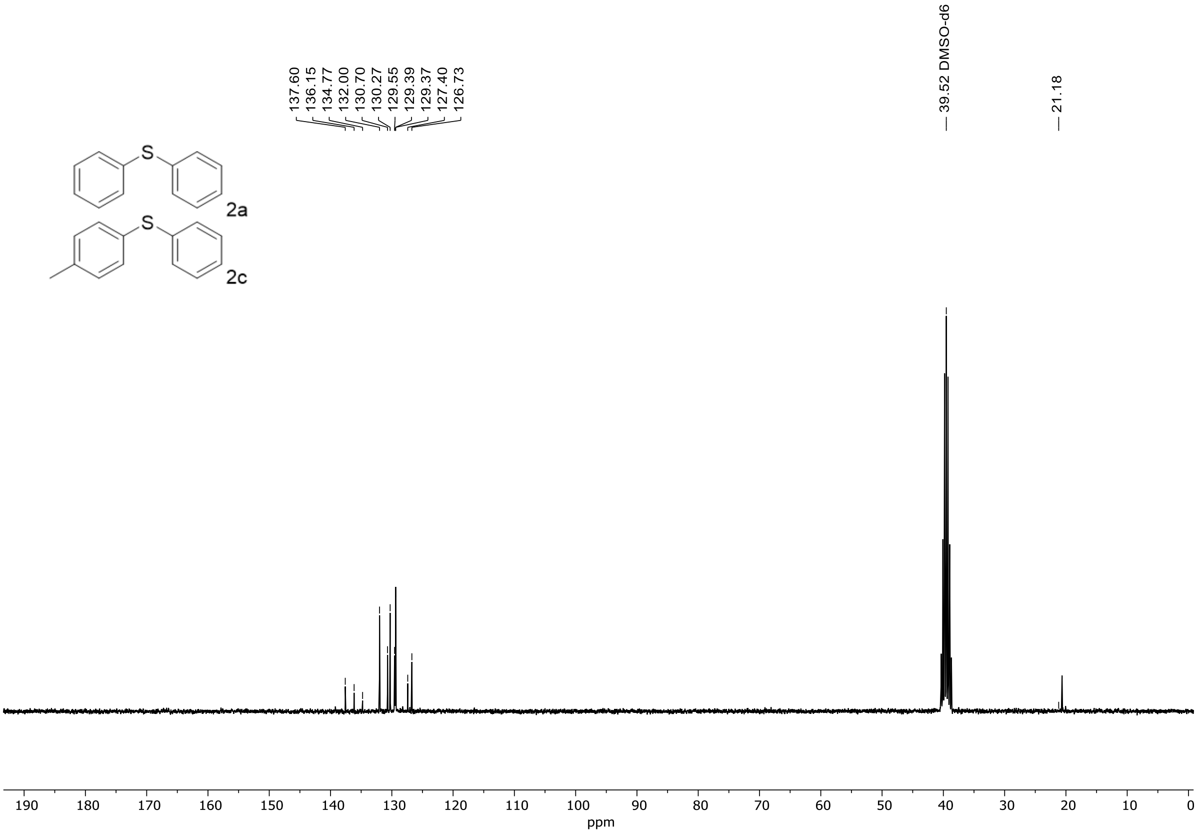

Diphenyl sulfide (2a), (4-chlorophenyl)(phenyl)sulfane (2d)

^{1}H NMR (300 MHz)

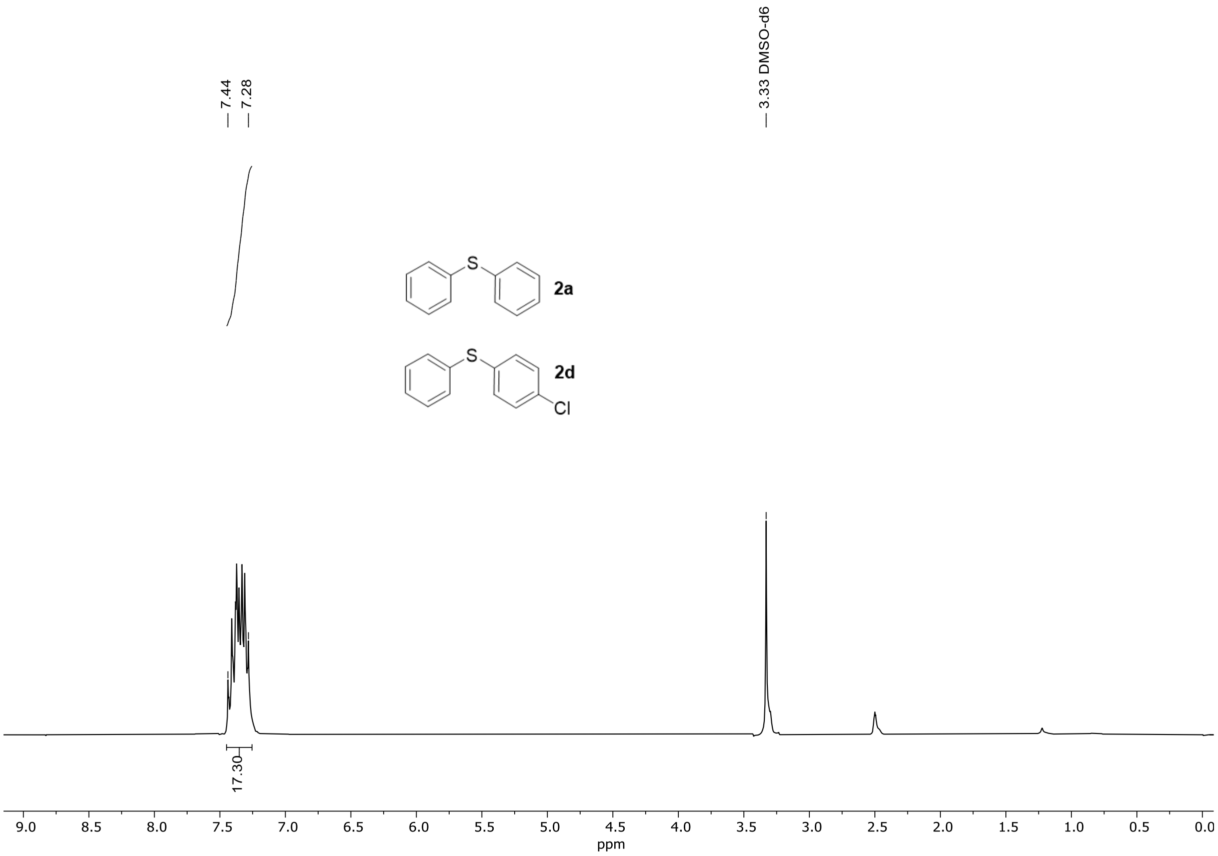

^{13}C NMR (75 MHz)

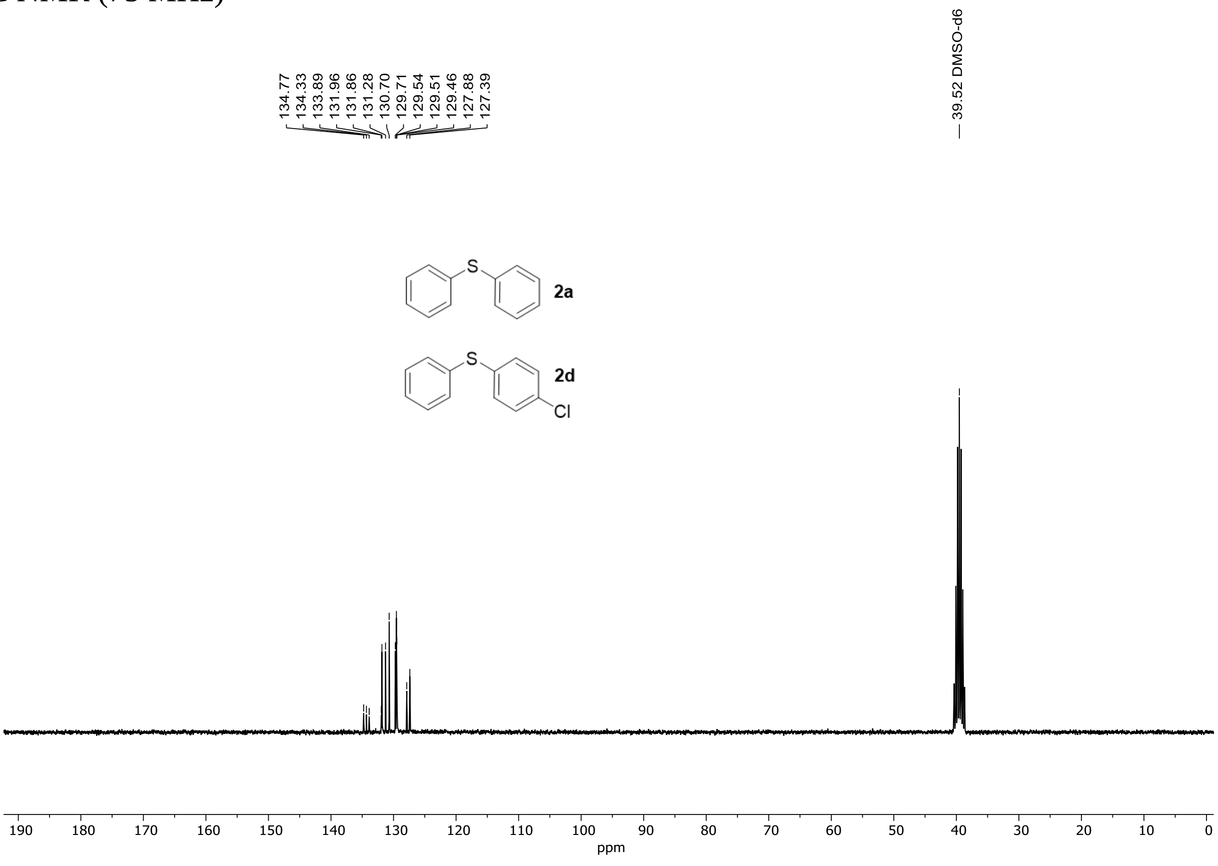

Supplement: File 1 — Experimental details, photophysics of CDs, cyclic voltammetry of triarylsulfonium salts and NMR spectra. [file Beilstein_J_Org_Chem-21-1024-s001.pdf]
